# Some series of intuitionistic fuzzy interactive averaging aggregation operators

**DOI:** 10.1186/s40064-016-2591-9

**Published:** 2016-07-07

**Authors:** Harish Garg

**Affiliations:** School of Mathematics, Thapar University Patiala, Patiala, Punjab 147004 India

**Keywords:** MCDM, Intuitionistic fuzzy set, Aggregation operator, Hamacher operation laws

## Abstract

In this paper, some series of new intuitionistic fuzzy averaging aggregation operators has been presented under the intuitionistic fuzzy sets environment. For this, some shortcoming of the existing operators are firstly highlighted and then new operational law, by considering the hesitation degree between the membership functions, has been proposed to overcome these. Based on these new operation laws, some new averaging aggregation operators namely, intuitionistic fuzzy Hamacher interactive weighted averaging, ordered weighted averaging and hybrid weighted averaging operators, labeled as IFHIWA, IFHIOWA and IFHIHWA respectively has been proposed. Furthermore, some desirable properties such as idempotency, boundedness, homogeneity etc. are studied. Finally, a multi-criteria decision making method has been presented based on proposed operators for selecting the best alternative. A comparative concelebration between the proposed operators and the existing operators are investigated in detail.

## Background

MCDM is one of the process for finding the optimal alternative from the set of feasible alternatives according to some criteria. Traditionally, it has been generally assumed that all the information which access the alternative in terms of criteria and their corresponding weights are expressed in the form of crisp numbers. But in day-today life, uncertainties play a crucial role in the decision making process. Due to complexities of the system, the decision maker may give their preferences corresponding to each alternative to some certain degree. However, it is obvious that much knowledge in the real world is fuzzy rather than precise and thus their corresponding analysis contains a lot of uncertainties and hence does not give the correct information to the practicing. Such kind of situations is suitably expressed with intuitionistic fuzzy sets (IFSs) (Attanassov 1986) rather than exact numerical values. These days IFSs are one of the most permissible theories to handle the uncertainties and impreciseness in the data than the crisp or probability theory (Garg 2013, 2016a, d; Garg et al. 2014; He et al. 2014b; Li and Nan 2009; Wan et al. 2016a; Xu 2007a, b; Yu 2015a). In the field of MCDM, the primary objective is of the information aggregation process. For this, Yager (1988) proposed the ordered weighted average (OWA) operator by giving some weights to all the inputs according to their ranking positions. Based on its pioneer work, many extensions have been appearing over it and applied it to solve the problems of multi-criteria decision making problems. For instance, Xu and Yager (2006) developed some geometric and Xu (2007a) proposed averaging aggregation operators on IFSs environment including weighted, ordered weighted and hybrid weighted operators. Zhao et al. (2010) combined Xu and Yager’s operators and developed their corresponding generalized aggregation operators. Xia and Xu (2010) proposed a series of intuitionistic fuzzy point aggregation operators based on the generalized aggregation operators (Zhao et al. 2010). He et al. (2014a) proposed an operations based on the principle of probability membership, non-membership and probability heterogenous functions operators. Wang and Liu (2011) and Wang and Liu (2012) proposed some geometric as well as averaging aggregation operator based on weighted and ordered weighted operators for different IFNs under Einstein operations. Zhao and Wei (2013) extended their aggregation operators by using the hybrid average and geometric operators. Apart from them, the various authors have addressed the problem of MCDM by using the different aggregation operators (Fei 2015; Garg 2015, 2016a, b, c, e; Garg et al. 2015; Liu 2014; Li and Ren 2015; Li and Wan 2014; Li 2014; Nan et al. 2016; Robinson and Amirtharaj 2015; Wan and Dong 2015; Wan et al. 2016a, b; Wang and Liu 2011; Xu and Yager 2006; Yu 2013a, b, 2015b; Yu and Shi 2015; Zhou et al. 2012).

It has been observed from the above aggregator operators that they have some drawbacks. For example, if there is an IFS whose at least one grade of non-membership function is zero, then the aggregated IFSs corresponding to the aggregator operators as described by Liu (2014), Wang and Liu (2011, 2013), Xu (2007a), Zhang and Yu (2014), Zhao et al. (2014) etc., have a zero degree of non-membership. This means that the role of the other grades of non-zero non-membership functions does not play any dominant role during the aggregation process. Similarly, if there is at least one degree of membership function to be zero then their corresponding IFSs obtained through geometric aggregator operators have a zero degree of membership functions. In other words, we can say that the effects of the other grades of either membership or non-membership on a corresponding geometric or an averaging aggregator operator does not play any significant role during the aggregation process. Further, it has been observed from above operators that the grades of overall membership (non-membership) functions are independent of their corresponding grades of non-membership (membership) functions. Thus, under such circumstances, the results corresponding to these operators are undesirable and hence does not give the reasonable preference order of the alternative.

Thus the objective of this manuscript is to present some new averaging aggregation operators under the IFSs environment. For this, some new operational laws on IFSs has been defined by considering the degree of hesitation between the grades of membership functions. Based on it, some series of different averaging aggregating operators including weighted average, ordered weighted averaging and hybrid weighted averaging have been proposed. It has been observed from these operators that the existing operators can be deduce from the proposed operators by giving a parameters to be a special numbers. Finally, a MCDM method based on these proposed aggregation operators are presented to show the applicability, utility and validity of the proposed ones. From the studies, it has been concluded that it can properly handle the shortcoming of the existing work and hence give an alternative way to finding the best alternative using an aggregation operators.

## Preliminaries

### Intuitionistic fuzzy set

An intuitionistic fuzzy set (IFS) *A* in a finite universe of discourse $X=\{x_1,x_2,\ldots ,x_n\}$ is given by (Attanassov 1986)

1 $$\begin{aligned} A = \left\{ \left\langle x, \mu _A(x), \nu _A(x)\right\rangle \mid x \in X \right\} \end{aligned}$$

where $\mu _A, \nu _A : X \longrightarrow [0, 1]$, respectively, be the membership and non-membership degree of the element *x* to the set *A* with the conditions $0 \le \mu _A(x), \nu _A(x)\le 1$, and $\mu _A(x)+\nu _A(x) \le 1$. For convenience, the pair $A=\langle \mu _A, \nu _A\rangle$ is called an intuitionistic fuzzy number (IFN) (Xu 2007a). Based on it, a score and accuracy function is defined as $S(A) = \mu _A - \nu _A$ and $H(A)=\mu _A + \nu _A$, respectively. In order to compare two two IFNs, $A_1 = \langle \mu _1, \nu _1\rangle$ and $A_2 = \langle \mu _2, \nu _2\rangle$, an order relation between them are summarized as follows (Wang et al. 2009; Xu 2007a).

(i) If $$S(A_1) > S(A_2)$$ then $$A_1 \succ A_2$$.
(ii) If $S(A_1) = S(A_2)$ then
  If $$H(A_1) > H(A_2)$$ then $$A_1 \succ A_2$$;
  If $$H(A_1) = H(A_2)$$ then $$A_1 = A_2$$.

### t-norm and t-conorm

t-norm (*T*) and t-conorm ($T^*$) operations are widely used for finding the various arithmetic operations in the IFSs environment. For instance, Xu (2007a) defined the algebraic product, sum, scalar and power operations for three IFNs $\alpha =\langle \mu , \nu \rangle$, $\alpha _1=\langle \mu _1,\nu _1\rangle$ and $\alpha _2=\langle \mu _2, \nu _2\rangle$ and $\lambda >0$ be a real number, by using t-norm ($T(x,y)=xy$) and t-cornorm ($T^*(x,y)=x+y-xy$) as follows

- $$\alpha _1 \oplus \alpha _2 = \langle 1-(1-\mu _1)(1-\mu _2), \nu _1\nu _2\rangle$$
- $$\alpha _1 \otimes \alpha _2 = \langle \mu _1\mu _2, 1-(1-\nu _1)(1-\nu _2)\rangle$$
- $$\lambda \alpha = \langle 1-(1-\mu )^{\lambda }, \nu ^{\lambda }\rangle$$
- $$\alpha ^{\lambda }=\langle \mu ^{\lambda }, 1-(1-\nu )^{\lambda }\rangle$$

On the other hand, if we define $T(x,y)=\frac{xy}{1+(1-x)(1-y)}$ and $T^*(x,y)=\frac{x+y}{1+xy}$ then the operations on IFN are known as Einstein t-norm and t-conorm respectively which are defined as below (Wang and Liu 2012)

- $$\alpha _1 \otimes \alpha _2 = \left\langle \dfrac{\mu _1\mu _2}{1+(1-\mu _1)(1-\mu _2)}, \dfrac{\nu _1 +\nu _2}{1+\nu _1\nu _2} \right\rangle$$
- $$\alpha _1 \oplus \alpha _2 = \left\langle \dfrac{\mu _1 +\mu _2}{1+\mu _1\mu _2}, \dfrac{\nu _1\nu _2}{1+(1-\nu _1)(1-\nu _2)} \right\rangle$$
- $$\lambda \alpha = \left\langle \dfrac{(1+\mu )^{\lambda }-(1-\mu )^{\lambda }}{(1+\mu )^{\lambda }+(1-\mu )^{\lambda }}, \dfrac{2 \nu ^{\lambda }}{(2-\nu )^{\lambda } + \nu ^{\lambda }} \right\rangle$$
- $$\alpha ^{\lambda } = \left\langle \dfrac{2 \mu ^{\lambda }}{(2-\mu )^{\lambda } + \mu ^{\lambda }}, \dfrac{(1+\nu )^{\lambda }-(1-\nu )^{\lambda }}{(1+\nu )^{\lambda }+(1-\nu )^{\lambda }}\right\rangle$$

Hamacher (1978) proposed a more generalized t-norm and t-conorm by defining as $T(x,y)=\frac{xy}{\gamma +(1-\gamma )(x+y-xy)}$ and $T^*(x,y)=\frac{x+y-xy-(1-\gamma )xy}{1-(1-\gamma )xy}$ respectively. It is clear from these operations that when $\gamma =1$ then they will reduce to algebraic t-norm and t-cornorm $T(x,y)=xy$ and $T^*(x,y)=x+y-xy$. Similarly when $\gamma =2$, they will reduce to Einstein t-norm and t-cornorm respectively as $T(x,y)=\frac{xy}{1+(1-x)(1-y)}$ and $T^*(x,y)=\frac{x+y}{1+xy}$. Thus, based on these operations, Hamacher sum and product operations are defined for two IFNs $\alpha _1$ and $\alpha _2$ as

- $$\alpha _1 \oplus \alpha _2 = \left\langle \dfrac{\mu _1+\mu _2-\mu _1\mu _2-(1-\gamma )\mu _1\mu _2}{1-(1-\gamma )\mu _1\mu _2} , \dfrac{\nu _1\nu _2}{\gamma +(1-\gamma )(\nu _1+\nu _2-\nu _1\nu _2)} \right\rangle$$
- $$\alpha _1\otimes \alpha _2 = \left\langle \dfrac{\mu _1\mu _2}{\gamma +(1-\gamma )(\mu _1+\mu _2-\mu _1\mu _2)}, \dfrac{\nu _1+\nu _2-\nu _1\nu _2-(1-\gamma )\nu _1\nu _2}{1-(1-\gamma )\nu _1\nu _2} \right\rangle$$

and their corresponding aggregation operators have been proposed by Liu (2014) for different IFNs $\alpha _i$’s by using weight vector $\omega =(\omega _1,\omega _2,\ldots ,\omega _n)^T$ of $\alpha _i (i=1,2,\ldots ,n)$ and $\omega _i >0$ and $\sum \nolimits _{i=1}^n \omega _i =1$ as

(i) The intuitionistic fuzzy Hamacher weighted averaging (IFHWA) operator
  $$\begin{aligned}&IFHWA(\alpha _1,\alpha _2,\ldots ,\alpha _n) = \omega _1 \alpha _1 \oplus \omega _2\alpha _2 \oplus \cdots \oplus \omega _n \alpha _n \\&\quad = \left\langle \frac{\prod \nolimits _{i=1}^n (1+(\gamma -1)\mu _i)^{\omega _i} - \prod \nolimits _{i=1}^n (1-\mu _i)^{\omega _i}}{\prod \nolimits _{i=1}^n (1+(\gamma -1)\mu _i)^{\omega _i} + (\gamma -1) \prod \nolimits _{i=1}^n (1-\mu _i)^{\omega _i}} , \frac{\gamma \prod \nolimits _{i=1}^n \nu _i^{\omega _i}}{\prod \nolimits _{i=1}^n (1+(\gamma -1)(1-\nu _i))^{\omega _i} + (\gamma -1) \prod \nolimits _{i=1}^n \nu _i^{\omega _i}}\right\rangle \end{aligned}$$
(ii) The intuitionistic fuzzy Hamacher ordered weighted averaging (IFHOWA) operator
  $$\begin{aligned}&IFHOWA(\alpha _1,\alpha _2,\ldots ,\alpha _n) = \omega _{\delta (1)}\alpha _{\delta (1)}\oplus \omega _{\delta (2)}\alpha _{\delta (2)} \oplus \cdots \oplus \omega _{\sigma (n)}\alpha _{\delta (n)} \\&\quad = \left\langle \frac{\prod \nolimits _{i=1}^n (1+(\gamma -1)\mu _{\delta (i)})^{\omega _i} - \prod \nolimits _{i=1}^n (1-\mu _{\delta (i)})^{\omega _i}}{\prod \nolimits _{i=1}^n (1+(\gamma -1)\mu _{\delta (i)})^{\omega _i} + (\gamma -1) \prod \nolimits _{i=1}^n (1-\mu _{\delta (i)})^{\omega _i}} , \frac{\gamma \prod \nolimits _{i=1}^n \nu _{\delta (i)}^{\omega _i}}{\prod \nolimits _{i=1}^n (1+(\gamma -1)(1-\nu _{\delta (i)}))^{\omega _i} + (\gamma -1) \prod \nolimits _{i=1}^n \nu _{\delta (i)}^{\omega _i}}\right\rangle \end{aligned}$$
  where $(\delta (1), \delta (2), \ldots , \delta (n))$ is a permutation of $(1,2,\ldots ,n)$ such that $\alpha _{\delta (i-1)} \ge \alpha _{\delta (i)}$ for all $i=1,2,\ldots ,n$.
(iii) The intuitionistic fuzzy Hamacher hybrid averaging (IFHHA) operator
  $$\begin{aligned}&IFHHA(\alpha _1,\alpha _2,\ldots ,\alpha _n) = \omega _{\sigma (1)}\dot{\alpha }_{\sigma (1)}\oplus \omega _{\sigma (2)}\dot{\alpha }_{\sigma (2)} \oplus \cdots \oplus \omega _{\sigma (n)}\dot{\alpha }_{\sigma (n)} \\&\quad = \left\langle \frac{\prod \nolimits _{i=1}^n (1+(\gamma -1)\dot{\mu }_{\sigma (i)})^{\omega _i} - \prod \nolimits _{i=1}^n (1-\dot{\mu }_{\sigma (i)})^{\omega _i}}{\prod \nolimits _{i=1}^n (1+(\gamma -1)\dot{\mu }_{\sigma (i)})^{\omega _i} + (\gamma -1) \prod \nolimits _{i=1}^n (1-\dot{\mu }_{\sigma (i)})^{\omega _i}} , \frac{\gamma \prod \nolimits _{i=1}^n \dot{\nu }_{\sigma (i)}^{\omega _i}}{\prod \nolimits _{i=1}^n (1+(\gamma -1)(1-\dot{\nu }_{\sigma (i)}))^{\omega _i} + (\gamma -1) \prod \nolimits _{i=1}^n \dot{\nu }_{\sigma (i)}^{\omega _i}}\right\rangle \end{aligned}$$
  where $\dot{\alpha }_{\sigma (i)}$ is the *i*th largest of the weighted intuitionistic fuzzy values $\dot{\alpha }_i$ ($\dot{\alpha }_i = n w_i \alpha _i, \,i=1,2,\ldots ,n)$.

The above operations are very concise and have been widely used by the various authors (He et al. 2014a, b; Liu 2014; Wang and Liu 2012; Xu 2007a; Zhao et al. 2010), but the above operations have several drawbacks. Few of them have listed as below.

#### *Example 1*

Let $$\alpha _1=\langle 0.72, 0\rangle$$, $$\alpha _2=\langle 0.55, 0.35\rangle$$, $$\alpha _3 = \langle 0.23, 0.72\rangle$$, $$\alpha _4 = \langle 0.33, 0.58\rangle$$ be four IFNs and $$\omega =(0.2, 0.3$$, $$0.4, 0.1)^T$$ is the standardized weight vector corresponding to these IFNs. By utilizing the IFHWA operator to aggregate all these numbers corresponding to $$\gamma =1$$ we get $$IFHWA(\alpha _1,\alpha _2,\alpha _3,\alpha _4)=\langle 0.4720, 0 \rangle$$ and for $$\gamma =2$$, we get IFHWA $$(\alpha _1,\alpha _2,\alpha _3,\alpha _4)$$$$=\langle 0.4582,0\rangle$$. From these results it has been seen that the degree of non-membership is zero and is independent of the parameter $$\gamma$$. Furthermore, this degree is independent of the degree of other non-membership (those which are nonzero in $$\alpha _i$$’s) and hence these plays an insignificant role during the aggregation process.

#### *Example 2*

Let $$\alpha _1=\langle 0.23, 0.35 \rangle$$, $$\alpha _2=\langle 0.45, 0.23\rangle$$, $$\alpha _3=\langle 0.65, 0.17 \rangle$$ and $$\alpha _4=\langle 0.50, 0.20\rangle$$ be four IFNs and $$\omega =(0.2, 0.3, 0.4$$, $$0.1)^T$$ is the standardized weight vector of these numbers. Then based on IFHWA operator we get the aggregated IFNs are $$\langle 0.5137, 0.2186\rangle$$ by taking $$\gamma =1$$ and $$\langle 0.5060, 0.2196\rangle$$ when $$\gamma =2$$. On the other hand, if we replace $$\alpha _2$$ and $$\alpha _3$$ IFNs with $$\beta _2=\langle 0.32, 0.23\rangle$$ and $$\beta _3 = \langle 0.37, 0.17\rangle$$ then their corresponding aggregated IFN become $$\langle 0.3443, 0.2186\rangle$$ when $$\gamma =1$$ and $$\langle 0.3422, 0.2196 \rangle$$ when $$\gamma =2$$. Hence, it has been seen that the degree of non-membership values of aggregated IFN becomes independent of the change of the degree of membership values. Therefore, it is inconsistent and hence does not give a correct information to the decision maker.

Therefore, the existing operators, as proposed by Liu (2014) are invalid to rank the alternative and hence there is a need to pay more attention on these issues.

## Some improved weighted averaging aggregator operators

In this section, we have define some improved aggregation operator by using an improved operational laws defined as below.

### **Definition 1**

Let $\alpha = \langle \mu , \nu \rangle$ and $\alpha _1=\langle \mu _1, \nu _1\rangle$, $\alpha _2 = \langle \mu _2, \nu _2 \rangle$ be three IFNs and $\lambda >0$ be a real number then some basic arithmetic operations between them have been defined by using Hamacher norms as follows

(i) $$\alpha _1 \oplus \alpha _2 = \left\langle \dfrac{\prod \nolimits _{i=1}^2 \left[ 1+(\gamma -1)\mu _i \right] -\prod \nolimits _{i=1}^2 (1-\mu _i)}{\prod \nolimits _{i=1}^2 \left[ 1+(\gamma -1)\mu _i \right] +(\gamma -1)\prod \nolimits _{i=1}^2 (1-\mu _i)}, \dfrac{\gamma \prod \nolimits _{i=1}^2 (1-\mu _i)-\gamma \prod \nolimits _{i=1}^2 \left[ 1-\mu _i-\nu _i\right] }{\prod \nolimits _{i=1}^2 \left[ 1+(\gamma -1)\mu _i \right] +(\gamma -1)\prod \nolimits _{i=1}^2 (1-\mu _i)} \right\rangle$$
(ii) $$\alpha _1 \otimes \alpha _2= \left\langle \dfrac{\gamma \prod \nolimits _{i=1}^2 (1-\nu _i)-\gamma \prod \nolimits _{i=1}^2 \left[ 1-\mu _i-\nu _i\right] }{\prod \nolimits _{i=1}^2 \left[ 1+(\gamma -1)\nu _i \right] +(\gamma -1)\prod \nolimits _{i=1}^2 (1-\nu _i)}, \quad \dfrac{\prod \nolimits _{i=1}^2 \left[ 1+(\gamma -1)\nu _i \right] -\prod \nolimits _{i=1}^2 (1-\nu _i)}{\prod \nolimits _{i=1}^2 \left[ 1+(\gamma -1)\mu _i \right] +(\gamma -1)\prod \nolimits _{i=1}^2 (1-\nu _i)} \right\rangle$$
(iii) $$\lambda \alpha = \left\langle \dfrac{\left[ 1+(\gamma -1)\mu \right] ^{\lambda }-\left[ 1-\mu \right] ^{\lambda }}{\left[ 1+(\gamma -1)\mu \right] ^{\lambda }+(\gamma -1)\left[ 1-\mu \right] ^{\lambda }}, \quad \dfrac{\gamma \left[ 1-\mu \right] ^{\lambda }-\gamma \left[ 1-\mu -\nu \right] ^{\lambda }}{\left[ 1+(\gamma -1)\mu \right] ^{\lambda } + (\gamma -1)\left[ 1-\mu \right] ^{\lambda }} \right\rangle$$
(iv) $$\alpha ^{\lambda } = \left\langle \dfrac{\gamma \left[ 1-\nu \right] ^{\lambda }-\gamma \left[ 1-\mu -\nu \right] ^{\lambda }}{ \left[ 1+(\gamma -1)\nu \right] ^{\lambda } + (\gamma -1)\left[ 1-\nu \right] ^{\lambda }}, \quad \dfrac{\left[ 1+(\gamma -1)\nu \right] ^{\lambda }- \left[ 1-\nu \right] ^{\lambda }}{\left[ 1+(\gamma -1)\mu \right] ^{\lambda }+(\gamma -1)\left[ 1-\nu \right] ^{\lambda }} \right\rangle$$

### Weighted average aggregation operator

#### **Definition 2**

Let $\varOmega$ is the set of IFNs $\alpha _i=\langle \mu _i, \nu _i\rangle , (i=1,2,\ldots ,n)$ and $\omega =(\omega _1,\omega _2,\ldots ,\omega _n)^T$ be its weight vector such that $\omega _i >0$ and $\sum \nolimits _{i=1}^n \omega _i=1$, and $IFHIWA: \varOmega ^n \longrightarrow \varOmega$, if

$$\begin{aligned} IFHIWA(\alpha _1,\alpha _2,\ldots ,\alpha _n)=\omega _1\alpha _1 \oplus \omega _2\alpha _2 \oplus \cdots \oplus \omega _n\alpha _n \end{aligned}$$

then IFHIWA is called an intuitionistic fuzzy Hamacher interactive weighting averaging operator.

#### **Theorem 1**

*Let*$\alpha _i=\langle \mu _i,\nu _i\rangle , (i=1,2,\ldots ,n)$*be the collection of IFNs, then*

2 $$\begin{aligned} IFHIWA(\alpha _1,\alpha _2,\ldots ,\alpha _n)= \left\langle \frac{\prod \nolimits _{i=1}^n (1+(\gamma -1)\mu _i)^{\omega _i} - \prod \nolimits _{i=1}^n (1-\mu _i)^{\omega _i}}{\prod \nolimits _{i=1}^n (1+(\gamma -1)\mu _i)^{\omega _i} + (\gamma -1)\prod \nolimits _{i=1}^n (1-\mu _i)^{\omega _i}} , \frac{\gamma \left\{ \prod \nolimits _{i=1}^n (1-\mu _i)^{\omega _i} - \prod \nolimits _{i=1}^n(1-\mu _i-\nu _i)^{\omega _i}\right\} }{\prod \nolimits _{i=1}^n (1+(\gamma -1)\mu _i)^{\omega _i} + (\gamma -1)\prod \nolimits _{i=1}^n (1-\mu _i)^{\omega _i}} \right\rangle \end{aligned}$$

#### *Proof*

When $n=1$ then $\omega =\omega _1=1$, and hence

$$\begin{aligned} IFHIWA(\alpha _1)=\omega _1 \alpha _1 = \langle \mu _1, \nu _1\rangle = \left\langle \frac{(1+(\gamma -1)\mu _1)^1 - (1-\mu _1)^1}{(1+(\gamma -1)\mu _1)^1 + (\gamma -1)(1-\mu _1)^1}, \frac{\gamma \left\{ (1-\mu _1)^1-(1-\mu _1-\nu _1)^1\right\} }{(1+(\gamma -1)\mu _1)^1 + (\gamma -1)(1-\mu _1)^1} \right\rangle \end{aligned}$$

Thus, results hold for $n=1$. Assume that result holds for $n=k$, i.e.,

$$\begin{aligned} IFHIWA(\alpha _1,\alpha _2,\ldots ,\alpha _k)= \left\langle \dfrac{\prod \nolimits _{i=1}^k (1+(\gamma -1)\mu _i)^{\omega _i}-\prod \nolimits _{i=1}^k(1-\mu _i)^{\omega _i}}{\prod \nolimits _{i=1}^k(1+(\gamma -1)\mu _i)^{\omega _i}+ (\gamma -1) \prod \nolimits _{i=1}^k(1-\mu _i)^{\omega _i}}, \frac{\gamma \left\{ \prod \nolimits _{i=1}^k(1-\mu _i)^{\omega _i}-\prod \nolimits _{i=1}^k (1-\mu _i-\nu _i)^{\omega _i} \right\} }{ \prod \nolimits _{i=1}^k(1+(\gamma -1) \mu _i)^{\omega _i}+(\gamma -1)\prod \nolimits _{i=1}^k(1-\mu _i)^{\omega _i}} \right\rangle \end{aligned}$$

By using the operational laws as given in Definition 1 for $n=k+1$ we have

$$\begin{aligned}&IFHIWA(\alpha _1,\alpha _2,\ldots ,\alpha _{k+1}) = \bigoplus _{i=1}^{k+1} \omega _i \alpha _i = IFHIWA(\alpha _1,\alpha _2,\ldots ,\alpha _{k}) \oplus \omega _{k+1} \alpha _{k+1} \\&\quad = \left\langle \dfrac{\prod \nolimits _{i=1}^k (1+(\gamma -1)\mu _i)^{\omega _i} - \prod \nolimits _{i=1}^k(1-\mu _i)^{\omega _i}}{\prod \nolimits _{i=1}^k(1+(\gamma -1)\mu _i)^{\omega _i} + (\gamma -1) \prod \nolimits _{i=1}^k(1-\mu _i)^{\omega _i}}, \frac{\gamma \left\{ \prod \nolimits _{i=1}^k(1-\mu _i)^{\omega _i}-\prod \nolimits _{i=1}^k (1-\mu _i-\nu _i)^{\omega _i} \right\} }{\prod \nolimits _{i=1}^k (1+(\gamma -1)\mu _i)^{\omega _i}+(\gamma -1)\prod \nolimits _{i=1}^k(1-\mu _i)^{\omega _i}} \right\rangle \\&\quad \oplus \left\langle \frac{(1+(\gamma -1)\mu _{k+1})^{\omega _{k+1}}-(1-\mu _{k+1})^{k+1}}{(1+(\gamma -1)\mu _{k+1})^{\omega _{k+1}}+(\gamma -1)(1-\mu _{k+1})^{k+1}}, \frac{\gamma \left\{ (1-\mu _{k+1})^{\omega _{k+1}} - (1-\mu _{k+1}-\nu _{k+1})^{\omega _{k+1}}\right\} }{(1+(\gamma -1)\mu _{k+1})^{\omega _{k+1}}+(\gamma -1)(1-\mu _{k+1})^{k+1}}\right\rangle \\&\quad = \left\langle \frac{\prod \nolimits _{i=1}^{k+1} (1+(\gamma -1)\mu _i)^{\omega _i} - \prod \nolimits _{i=1}^{k+1} (1-\mu _i)^{\omega _i}}{\prod \nolimits _{i=1}^{k+1} (1+(\gamma -1)\mu _i)^{\omega _i} + (\gamma -1)\prod \nolimits _{i=1}^{k+1} (1-\mu _i)^{\omega _i}} , \frac{\gamma \left\{ \prod \nolimits _{i=1}^{k+1} (1-\mu _i)^{\omega _i} - \prod \nolimits _{i=1}^{k+1}(1-\mu _i-\nu _i)^{\omega _i}\right\} }{\prod \nolimits _{i=1}^{k+1} (1+(\gamma -1)\mu _i)^{\omega _i} + (\gamma -1) \prod \nolimits _{i=1}^{k+1} (1-\mu _i)^{\omega _i}} \right\rangle \end{aligned}$$

Hence complete the proof. $\square$

#### **Lemma 1**

(Xu 2007a) *Let*$\alpha _i=\langle \mu _i, \nu _i\rangle , \omega _i>0$*for*$i=1,2,\ldots ,n$*and*$\sum \nolimits _{i=1}^n \omega _i=1$, *then*

$$\begin{aligned} \prod \limits _{i=1}^n \alpha _i ^{\omega _i} \le \sum \limits _{i=1}^n \omega _i \alpha _i \end{aligned}$$

*with equality holds if and only if*$\alpha _1 = \alpha _2=\cdots =\alpha _n$.

#### **Corollary 1**

*Let*$\alpha _i, (i=1,2,\ldots ,n)$*be a collections of IFNs then the operators IFHWA and IFHIWA have the following relation:*

$$\begin{aligned} IFHIWA(\alpha _1,\alpha _2,\ldots ,\alpha _n)\le IFHWA(\alpha _1,\alpha _2,\ldots ,\alpha _n) \end{aligned}$$

#### *Proof*

Let $IFHIWA(\alpha _1,\alpha _2,\ldots ,\alpha _n)=\langle \mu _{\alpha }^p , \nu _{\alpha }^p\rangle = \alpha ^p$ and $IFHWA(\alpha _1,\alpha _2,\ldots ,\alpha _n)=\langle \mu _{\alpha } , \nu _{\alpha }\rangle = \alpha$, and $\omega =(\omega _1,\omega _2,\ldots ,\omega _n)^T$ be its corresponding weight vectors then

$$\begin{aligned}&\prod \limits _{i=1}^n (1+(\gamma -1)\mu _i)^{\omega _i} + (\gamma -1)\prod \limits _{i=1}^n (1-\mu _i)^{\omega _i} \le \sum \limits _{i=1}^n \omega _i (1+(\gamma -1)\mu _i) + (\gamma -1)\sum \limits _{i=1}^n \omega _i(1-\mu _i) = \gamma \end{aligned}$$

and

$$\begin{aligned} \nu _{\alpha }^p &= \dfrac{\gamma \left\{ \prod \nolimits _{i=1}^{n}(1-\mu _i)^{\omega _i}-\prod \nolimits _{i=1}^{n} (1-\mu _i-\nu _i)^{\omega _i}\right\} }{ \prod \nolimits _{i=1}^{n}(1+(\gamma -1)\mu _i)^{\omega _i}+(\gamma -1)\prod \nolimits _{i=1}^{n}(1-\mu _i)^{\omega _i}} \\ &\ge {} \prod \limits _{i=1}^{n} (1-\mu _i)^{\omega _i} -\prod \limits _{i=1}^{n} (1-\mu _i-\nu _i)^{\omega _i} \\ & \ge {} \dfrac{\gamma \prod \nolimits _{i=1}^{n} \nu _i^{\omega _i}}{\prod \nolimits _{i=1}^{n} (1+(\gamma -1)(1-\nu _i))^{\omega _i}+(\gamma -1)\prod \nolimits _{i=1}^{n} \nu _i^{\omega _i}} = \nu _\alpha \end{aligned}$$

Thus, $\nu _{\alpha }^p \ge \nu _{\alpha }$ where equality holds if and only if $\mu _1=\mu _2=\cdots =\mu _n$ and $\nu _1=\nu _2=\cdots =\nu _n$.

Therefore,

$$\begin{aligned} S(\alpha ^p)=\mu _{\alpha }^p - \nu _{\alpha }^p \le \mu _{\alpha }-\nu _{\alpha } = S(\alpha ) \end{aligned}$$

If $S(\alpha ^p) < S(\alpha )$ then for every $\omega$, we have

$$\begin{aligned} IFHIWA(\alpha _1,\alpha _2,\ldots ,\alpha _n) < IFHWA(\alpha _1,\alpha _2,\ldots ,\alpha _n) \end{aligned}$$

If $S(\alpha ^p) = S(\alpha )$ i.e. $\mu _\alpha ^p-\nu _\alpha ^p= \mu _\alpha -\nu _\alpha$ then by the condition $\nu _\alpha ^p \ge \nu _\alpha$, we have $\mu _\alpha ^p=\mu _\alpha$ and $\nu _\alpha ^p = \nu _\alpha$, thus the accuracy function $H(\alpha ^p)=\mu _\alpha ^p + \nu _\alpha ^p=\mu _\alpha + \nu _\alpha = H(\alpha )$. Thus in this case, from the definition of score function, it follows that

$$\begin{aligned} IFHIWA(\alpha _1,\alpha _2,\ldots ,\alpha _n) = IFHWA(\alpha _1,\alpha _2,\ldots ,\alpha _n) \end{aligned}$$

Hence,

$$\begin{aligned} IFHIWA(\alpha _1,\alpha _2,\ldots ,\alpha _n) \le IFHWA(\alpha _1,\alpha _2,\ldots ,\alpha _n) \end{aligned}$$

where that equality holds if and only if $\alpha _1=\alpha _2=\cdots = \alpha _n$. $\square$

From this corollary it has been concluded that the proposed IFHIWA operator shows the decision maker’s more optimistic attitude than the existing IFHWA operator (Liu 2014) in aggregation process.

#### *Example 3*

Let $$\alpha _1=\langle 0.1, 0.7\rangle , \alpha _2=\langle 0.4, 0.3\rangle , \alpha _3=\langle 0.6, 0.1\rangle$$ and $$\alpha _4=\langle 0.2,0.5\rangle$$ be four IFNs and $$\omega =(0.2,0.3,0.1,0.4)^T$$ be the weight vector of $$\alpha _i$$’s, i.e. $$\mu _{1}=0.1$$, $$\mu _{2}=0.4,$$$$\mu _{3}=0.6$$, $$\mu _{4}=0.2$$, $$\nu _{1}=0.7$$, $$\nu _{2}=0.3$$, $$\nu _{3}=0.1$$, $$\nu _{4}=0.5$$; then for $$\gamma =2$$, we have

$$\begin{aligned} IFHIWA(\alpha _1, \alpha _2,\alpha _3,\alpha _4) &= \left\langle \frac{\prod \nolimits _{i=1}^4(1+\mu _i)^{\omega _i} - \prod \nolimits _{i=1}^4 (1-\mu _i)^{\omega _i}}{\prod \nolimits _{i=1}^4(1+\mu _i)^{\omega _i} + \prod \nolimits _{i=1}^4 (1-\mu _i)^{\omega _i}}, \frac{2\left\{ \prod \nolimits _{i=1}^4 (1-\mu _i)^{\omega _i}-\prod \nolimits _{i=1}^n (1-\mu _i-\nu _i)^{\omega _i}\right\} }{\prod \nolimits _{i=1}^4(1+\mu _i)^{\omega _i} + \prod \nolimits _{i=1}^4 (1-\mu _i)^{\omega _i}} \right\rangle \\& =\left\langle \frac{1.2712 -0.7010}{1.2712 + 0.7010}, \frac{2\times (0.7010-0.2766)}{1.2712 + 0.7010}\right\rangle\\ & = \langle 0.2891, 0.4304\rangle \\ IFHWA(\alpha _1, \alpha _2,\alpha _3,\alpha _4) & = \left\langle \frac{\prod \nolimits _{i=1}^4(1+\mu _i)^{\omega _i} - \prod \nolimits _{i=1}^4 (1-\mu _i)^{\omega _i}}{\prod \nolimits _{i=1}^4(1+\mu _i)^{\omega _i} + \prod \nolimits _{i=1}^4 (1-\mu _i)^{\omega _i}}, \frac{2\prod \nolimits _{i=1}^4 \nu _i^{\omega _i}}{\prod \nolimits _{i=1}^4(2-\nu _i)^{\omega _i} + \prod \nolimits _{i=1}^4 (\nu _i)^{\omega _i}} \right\rangle \\& = \left\langle \frac{1.2712 -0.7010}{1.2712 + 0.7010}, \frac{2\times 0.3906}{1.5497 + 0.3906} \right\rangle \\ & = \langle 0.2891, 0.4026\rangle \\ IFWA(\alpha _1,\alpha _2,\alpha _3,\alpha _4) & = \left\langle 1-\prod \nolimits _{i=1}^n (1-\mu _i)^{\omega _i}, \prod \nolimits _{i=1}^n (\nu _i)^{\omega _i}\right\rangle \\& = \langle 0.2990, 0.3906 \rangle \end{aligned}$$

Thus, it has been concluded that

$$\begin{aligned} S(IFHIWA)< S(IFHWA) < S(IFWA) \end{aligned}$$

#### **Theorem 2**

*If*$\alpha _i =\langle \mu _i, \nu _i\rangle$*be an IFNs*, $i=1,2,\ldots ,n$, *then the aggregated value by using IFHIWA operator is also an IFN i.e.*

$$\begin{aligned} IFHIWA(\alpha _1,\alpha _2,\ldots ,\alpha _n) \in IFN \end{aligned}$$

#### *Proof*

Since $\alpha _i = \langle \mu _i, \nu _i\rangle$ be an IFNs for $i=1,2,\ldots ,n$, then by definition of IFN, we have

$$\begin{aligned} 0 \le \mu _i, \nu _i \le 1 \quad \text{ and }\quad \mu _i+\nu _i \le 1 \end{aligned}$$

Take, $IFHIWA(\alpha _1,\ldots ,\alpha _n) = \langle \mu _{IFHIWA}, \nu _{IFHIWA}\rangle$, we have

$$\begin{aligned}&\frac{\prod \nolimits _{i=1}^n (1+(\gamma -1)\mu _i)^{\omega _i} - \prod \nolimits _{i=1}^n (1-\mu _i)^{\omega _i}}{\prod \nolimits _{i=1}^n (1+(\gamma -1)\mu _i)^{\omega _i} + (\gamma -1)(1-\mu _i)^{\omega _i}} = 1 - \frac{\gamma \prod \nolimits _{i=1}^n (1-\mu _i)^{\omega _i}}{\prod \nolimits _{i=1}^n (1+(\gamma -1)\mu _i)^{\omega _i} + (\gamma -1)\prod \limits _{i=1}^n (1-\mu _i)^{\omega _i}} \le 1 - \prod \nolimits _{i=1}^n (1-\mu _i)^{\omega _i} \le 1 \end{aligned}$$

Also

$$\begin{aligned} 1+(\gamma -1)\mu _i \ge (1-\mu _i)\Leftrightarrow & {} \prod \limits _{i=1}^n (1+(\gamma -1)\mu _i)^{\omega _i} - \prod \limits _{i=1}^n (1-\mu _i)^{\omega _i} \ge 0 \\\Leftrightarrow & {} \frac{\prod \nolimits _{i=1}^n(1+(\gamma -1)\mu _i)^{\omega _i} - \prod \nolimits _{i=1}^n (1-\mu _i)^{\omega _i}}{\prod \nolimits _{i=1}^n (1+(\gamma -1)\mu _i)^{\omega _i} + (\gamma -1)\prod \nolimits _{i=1}^n(1-\mu _i)^{\omega _i}} \ge 0. \end{aligned}$$

Thus $0 \le \mu _{IFHIWA} \le 1$. On the other hand,

$$\begin{aligned} \frac{\gamma \left\{ \prod \nolimits _{i=1}^n (1-\mu _i)^{\omega _i} - \prod \nolimits _{i=1}^n (1-\mu _i-\nu _i)^{\omega _i} \right\} }{\prod \nolimits _{i=1}^n (1+(\gamma -1)\mu _i)^{\omega _i} + (\gamma -1) \prod \nolimits _{i=1}^n(1-\mu _i)^{\omega _i}}\le & {} \frac{\gamma \prod \nolimits _{i=1}^n (1-\mu _i)^{\omega _i}}{\prod \nolimits _{i=1}^n (1+(\gamma -1) \mu _i)^{\omega _i} + (\gamma -1) \prod \nolimits _{i=1}^n(1-\mu _i)^{\omega _i}} \\\le & {} \prod \nolimits _{i=1}^n (1-\mu _i)^{\omega _i} \le 1 \qquad \mid \because \text {of Lemma }1 \end{aligned}$$

Also

$$\begin{aligned} \prod \limits _{i=1}^n (1-\mu _i)^{\omega _i} - \prod \limits _{i=1}^n (1-\mu _i-\nu _i)^{\omega _i} \ge 0 \Leftrightarrow \frac{\gamma \left\{ \prod \nolimits _{i=1}^n (1-\mu _i)^{\omega _i} - \prod \nolimits _{i=1}^n (1-\mu _i-\nu _i)^{\omega _i} \right\} }{\prod \nolimits _{i=1}^n (1+(\gamma -1) \mu _i)^{\omega _i} + (\gamma -1) \prod \nolimits _{i=1}^n(1-\mu _i)^{\omega _i}} \ge 0 \end{aligned}$$

Thus $$0 \le \nu _{IFHIWA} \le 1$$.

Finally,

$$\begin{aligned} \mu _{IFHIWA} + \nu _{IFHIWA} & = 1 - \frac{\gamma \prod \nolimits _{i=1}^n (1-\mu _i-\nu _i)^{\omega _i}}{\prod \nolimits _{i=1}^n (1+(\gamma -1)\mu _i)^{\omega _i} + (\gamma -1) \prod \limits _{i=1}^n (1-\mu _i)^{\omega _i}} \\ & \le {} 1 - \prod \nolimits _{i=1}^n (1-\mu _i-\nu _i)^{\omega _i} \le 1 \end{aligned}$$

Hence, $IFHIWA \in [0, 1]$. Therefore, the aggregated IFN obtained by IFHIWA operator is again an IFN. $\square$

#### *Example 4*

If we apply the proposed IFHIWA operator on Example 1 then corresponding to $\gamma =1$, we get the aggregated IFNs as $IFHIWA(\alpha _1,\alpha _2,\alpha _3,\alpha _4)=\langle 0.4720, 0.4358\rangle$ while for $\gamma =2$ we have $IFHIWA(\alpha _1,\alpha _2,\alpha _3,\alpha _4)=\langle 0.4582, 0.4473\rangle$. Therefore, it has been seen that there is a non-zero degree of non-membership of the overall aggregated IFNs even if at least one of their corresponding grades of IFNs is zero. Thus, the others grades of non-membership function of IFNs play a dominant role during the aggregation process in the proposed operator.

#### *Example 5*

If we apply the proposed IFHIWA operator to aggregate the different IFNs as given in Example 2 then we get aggregated IFN are $\langle 0.5137, 0.2196\rangle$ when $\gamma =1$ and $\langle 0.5060, 0.2231\rangle$ when $\gamma =2$. On the other hand, if we apply proposed aggregated operator on modified IFNs then we get $IFHIWA(\alpha _1,\beta _2,\beta _3,\alpha _4)=\langle 0.3443, 0.2257\rangle$ for $\gamma =1$ and $\langle 0.3422, 0.2264 \rangle$ for $\gamma =2$. Thus, the change of membership function will affect on the degree of non-membership functions and is non-zero. Therefore, there is a proper interaction between the degree of membership and non-membership functions and hence the results are consistent and more practical than the existing operators results.

Now, based on Theorem 1, we have some properties of the proposed IFHIWA operator for a collection of IFNs $\alpha _i = \langle \mu _i, \nu _i\rangle , (i=1,2,\ldots ,n)$ and $\omega = (\omega _1,\omega _2,\ldots ,\omega _n)^T$ is the associated weighted vector satisfying $\omega _i \in [0, 1]$ and $\sum \nolimits _{i=1}^n \omega _i =1$.

#### **Property 1**

(Idempotency) *If *$\alpha _i=\alpha _0 = \langle \mu _{0}, \nu _{0}\rangle$*for alli, then*

$$\begin{aligned} IFHIWA(\alpha _1,\alpha _2,\ldots ,\alpha _n)=\alpha _0 \end{aligned}$$

#### *Proof*

Since $\alpha _i = \alpha _0 = \langle \mu _{0}, \nu _{0}\rangle (i=1,2,\ldots ,n)$ and $\sum \nolimits _{i=1}^n \omega _i=1$, so by Theorem 1, we have

$$\begin{aligned} IFHIWA(\alpha _1,\alpha _2,\ldots ,\alpha _n) & = \left\langle \frac{\prod \nolimits _{i=1}^n (1+(\gamma -1)\mu _{0})^{\omega _i}-\prod \nolimits _{i=1}^n (1-\mu _{0})^{\omega _i}}{\prod \nolimits _{i=1}^n (1+(\gamma -1) \mu _{0})^{\omega _i} + (\gamma -1) \prod \nolimits _{i=1}^n (1-\mu _{0})^{\omega _i}}, \right. \\&\qquad \left. \frac{\gamma \left\{ \prod \nolimits _{i=1}^n (1-\mu _{0})^{\omega _i}-\prod \nolimits _{i=1}^n(1-\mu _{0}-\nu _{0})^{\omega _i} \right\} }{\prod \nolimits _{i=1}^n (1+(\gamma -1) \mu _{0})^{\omega _i} + (\gamma -1) \prod \nolimits _{i=1}^n (1-\mu _{0})^{\omega _i}} \right\rangle \\ &= \left\langle \frac{(1+(\gamma -1)\mu _{0})^{\sum \nolimits _{i=1}^n\omega _i}-(1-\mu _{0})^{\sum \nolimits _{i=1}^n\omega _i}}{(1+(\gamma -1) \mu _{0})^{\sum \nolimits _{i=1}^n \omega _i} + (\gamma -1)(1-\mu _{0})^{\sum \nolimits _{i=1}^n\omega _i}}, \right. \\&\qquad \qquad \left. \frac{\gamma \left\{ (1-\mu _{0})^{\sum \nolimits _{i=1}^n\omega _i} - (1-\mu _{0}-\nu _{0})^{\sum \nolimits _{i=1}^n\omega _i} \right\} }{(1+(\gamma -1)\mu _{0})^{\sum \nolimits _{i=1}^n\omega _i} + (\gamma -1)(1-\mu _{0})^{\sum \nolimits _{i=1}^n\omega _i}} \right\rangle \\ &= \left\langle \frac{(1+(\gamma -1)\mu _{0})-(1-\mu _{0})}{(1+(\gamma -1)\mu _{0})+(\gamma -1)(1-\mu _{0})}, \frac{\gamma \left\{ (1-\mu _{0})-(1-\mu _{0}-\nu _{0})\right\} }{(1+(\gamma -1)\mu _{0})+(\gamma -1)(1-\mu _{0})} \right\rangle \\ & = \langle \mu _{0}, \nu _{0} \rangle \\ & = \alpha _0 \end{aligned}$$

$$\square$$

#### **Property 2**

(Boundedness) *Let*$\alpha ^- = \langle \min \nolimits _i(\mu _{i}), \max \nolimits _i (\nu _{i}) \rangle$*and*$\alpha ^+ = \langle \max \nolimits _i(\mu _{i}), \min \nolimits _i(\nu _{i})\rangle$*then*

$$\begin{aligned} \alpha ^- \le IFHIWA(\alpha _1,\alpha _2,\ldots ,\alpha _n) \le \alpha ^+ \end{aligned}$$

#### *Proof*

Let $f(x)=\frac{1-x}{1+(\gamma -1)x}, x\in [0,1]$ then $f^{'}(x)=\frac{-\gamma }{(1+(\gamma -1)x)^2} < 0$; thus, *f*(*x*) is decreasing function. Since $\mu _{i,\min } \le \mu _i \le \mu _{i,\max }$, for all $i=1,2,\ldots ,n$ then $f(\mu _{i,\max }) \le f(\mu _i) \le f(\mu _{i,\min })$ for all *i*, i.e. $\frac{1-\mu _{i,\max }}{1+(\gamma -1)\mu _{i,\max }} \le \frac{1-\mu _i}{1+(\gamma -1)\mu _i} \le \frac{1-\mu _{i,\min }}{1+(\gamma -1)\mu _{i,\min }}$, for all *i*. Let $\omega = (\omega _1,\omega _2,\ldots ,\omega _n)^T$ is the associated weighted vector satisfying $\omega _i \in [0, 1]$ and $\sum \nolimits _{i=1}^n \omega _i =1$, then for all *i*, we have $\left( \dfrac{1-\mu _{i,\max }}{1+(\gamma -1)\mu _{i,\max }}\right) ^{\omega _i} \le \left( \dfrac{1-\mu _i}{1+(\gamma -1)\mu _i}\right) ^{\omega _i} \le \left( \dfrac{1-\mu _{i,\min }}{1+(\gamma -1)\mu _{i,\min }}\right) ^{\omega _i}$

Thus,

3 $$\begin{aligned}&\prod \limits _{i=1}^n \left( \frac{1-\mu _{i,\max }}{1+(\gamma -1)\mu _{i,\max }}\right) ^{\omega _i} \le \prod \limits _{i=1}^n \left( \frac{1-\mu _i}{1+(\gamma -1)\mu _i}\right) ^{\omega _i} \le \prod \limits _{i=1}^n \left( \frac{1-\mu _{i,\min }}{1+(\gamma -1)\mu _{i,\min }}\right) ^{\omega _i} \nonumber \\&\quad \Leftrightarrow (\gamma -1) \left( \frac{1-\mu _{i,\max }}{1+(\gamma -1)\mu _{i,\max }}\right) \le (\gamma -1) \prod \limits _{i=1}^n \left( \frac{1-\mu _i}{1+(\gamma -1)\mu _i}\right) ^{\omega _i} \le (\gamma -1) \left( \frac{1-\mu _{i,\min }}{1+(\gamma -1)\mu _{i,\min }}\right) \nonumber \\&\quad \Leftrightarrow \left( \frac{\gamma }{1+(\gamma -1)\mu _{i,\max }}\right) \le 1+(\gamma -1) \prod \limits _{i=1}^n \left( \frac{1-\mu _i}{1+(\gamma -1)\mu _i}\right) ^{\omega _i} \le \left( \frac{\gamma }{1+(\gamma -1)\mu _{i,\min }}\right) \nonumber \\&\quad \Leftrightarrow \left( \frac{1+(\gamma -1)\mu _{i,\min }}{\gamma }\right) \le \frac{1}{1+(\gamma -1)\prod \nolimits _{i=1}^n \left( \dfrac{1-\mu _i}{1+(\gamma -1)\mu _i}\right) ^{\omega _i}}\le \left( \frac{1+(\gamma -1)\mu _{i,\max }}{\gamma }\right) \nonumber \\&\quad \Leftrightarrow 1+(\gamma -1) \mu _{i,\min } \le \frac{\gamma }{1 + (\gamma -1) \prod \nolimits _{i=1}^n \left( \dfrac{1-\mu _i}{1+(\gamma -1)\mu _i}\right) ^{\omega _i}}\le 1+(\gamma -1)\mu _{i,\max } \nonumber \\&\quad \Leftrightarrow (\gamma -1) \mu _{i,\min } \le \frac{\gamma }{1+(\gamma -1)\prod \nolimits _{i=1}^n \left( \dfrac{1-\mu _i}{1 + (\gamma -1) \mu _i}\right) ^{\omega _i}} -1 \le (\gamma -1) \mu _{i,\max } \nonumber \\&\quad \Leftrightarrow \mu _{i,\min } \le \frac{\prod \nolimits _{i=1}^n (1+(\gamma -1)\mu _i)^{\omega _i} - \prod \nolimits _{i=1}^n (1-\mu _i)^{\omega _i}}{\prod \nolimits _{i=1}^n (1+(\gamma -1)\mu _i)^{\omega _i} + (\gamma -1)\prod \nolimits _{i=1}^n (1-\mu _i)^{\omega _i}} \le \mu _{i,\max } \end{aligned}$$

On the other hand, let $g(y)=\frac{\gamma -(\gamma -1)y}{(\gamma -1)y}, y \in [0,1]$ then $g^{\prime }(y)=-\gamma /((\gamma -1))^2y^2 <0$ so *g*(*y*) is decreasing function on (0,1]. Since $1-\mu _{i,\max } \le 1-\mu _i \le 1-\mu _{i,\min }$ for all *i* then $g(1-\mu _{i,\min })\le g(1-\mu _i) \le g(1-\mu _{i,\max })$ i.e. $\frac{\gamma -(\gamma -1)(1-\mu _{i,\min })}{(\gamma -1)(1-\mu _{i,\min })} \le \frac{\gamma -(\gamma -1)(1-\mu _i)}{(\gamma -1)(1-\mu _i)} \le \frac{\gamma -(\gamma -1)(1-\mu _{i,\max })}{(\gamma -1)(1-\mu _{i,\max })}$ for all $i=1,2,\ldots ,n$. Let $\omega = (\omega _1,\omega _2,\ldots ,\omega _n)^T$ is the associated weighted vector satisfying $\omega _i \in [0, 1]$ and $\sum \nolimits _{i=1}^n \omega _i =1$, then for all *i*, we have

$$\begin{aligned} \left( \dfrac{\gamma -(\gamma -1)(1-\mu _{i,\min })}{(\gamma -1)(1-\mu _{i,\min })}\right) ^{\omega _i} \le \left( \dfrac{\gamma -(\gamma -1)(1-\mu _i)}{(\gamma -1)(1-\mu _i)}\right) ^{\omega _i} \le \left( \dfrac{\gamma -(\gamma -1)(1-\mu _{i,\max })}{(\gamma -1)(1-\mu _{i,\max })}\right) ^{\omega _i} \end{aligned}$$

Thus,

$$\begin{aligned}&\prod \limits _{i=1}^n \left( \frac{\gamma -(\gamma -1)(1-\mu _{i,\min })}{(\gamma -1)(1-\mu _{i,\min })}\right) ^{\omega _i} \le \prod \limits _{i=1}^n \left( \frac{\gamma -(\gamma -1)(1-\mu _i)}{(\gamma -1)(1-\mu _i)}\right) ^{\omega _i} \le \prod \limits _{i=1}^n \left( \frac{\gamma -(\gamma -1)(1-\mu _{i,\max })}{(\gamma -1)(1-\mu _{i,\max })}\right) ^{\omega _i}\\&\quad \Leftrightarrow \frac{\gamma -(\gamma -1)(1-\mu _{i,\min })}{(\gamma -1)(1-\mu _{i,\min })}\le \prod \limits _{i=1}^n \left( \frac{\gamma -(\gamma -1)(1-\mu _i)}{(\gamma -1)(1-\mu _i)}\right) ^{\omega _i} \le \frac{\gamma -(\gamma -1)(1-\mu _{i,\max })}{(\gamma -1)(1-\mu _{i,\max })}\\&\quad \Leftrightarrow \frac{\gamma }{(\gamma -1)(1-\mu _{i,\min })} \le \prod \limits _{i=1}^n \left( \frac{\gamma -(\gamma -1)(1-\mu _i)}{(\gamma -1)(1-\mu _i)}\right) ^{\omega _i} + 1 \le \frac{\gamma }{(\gamma -1)(1-\mu _{i,\max })} \\&\quad \Leftrightarrow \frac{(\gamma -1)(1-\mu _{i,\max })}{\gamma } \le \frac{1}{\prod \nolimits _{i=1}^n \left( \dfrac{\gamma -(\gamma -1)(1-\mu _i)}{(\gamma -1) (1-\mu _i)}\right) ^{\omega _i} +1} \le \frac{(\gamma -1)(1-\mu _{i,\min })}{\gamma } \\&\quad \Leftrightarrow 1-\mu _{i,\max }\le \frac{\gamma }{(\gamma -1)\prod \nolimits _{i=1}^n \left( \dfrac{\gamma -(\gamma -1)\nu _i}{(\gamma -1)\nu _i}\right) ^{\omega _i} + (\gamma -1)} \le 1-\mu _{i,\min } \end{aligned}$$

Also

4 $$\begin{aligned}&1- \mu _{i,\max } - \nu _{i,\min } \le 1-\mu _i - \nu _i \le 1 - \mu _{i,\min } - \nu _{i,\max } \nonumber \\&\quad \Leftrightarrow \frac{1- \mu _{i,\max } - \nu _{i,\min }}{1-\mu _{i,\min }} \le \frac{1-\mu _i - \nu _i}{1-\mu _i} \le \frac{1 - \mu _{i,\min } - \nu _{i,\max }}{1-\mu _{i,\max }} \nonumber \\&\quad \Leftrightarrow \frac{1- \mu _{i,\max } - \nu _{i,\min }}{1-\mu _{i,\min }} \le \prod \limits _{i=1}^n \left( \frac{1-\mu _i - \nu _i}{1-\mu _i}\right) ^{\omega _i} \le \frac{1 - \mu _{i,\min } - \nu _{i,\max }}{1-\mu _{i,\max }} \nonumber \\&\quad \Leftrightarrow \frac{-\mu _{i,\max }+\mu _{i,\min }+\nu _{i,\max }}{1-\mu _{i,\max }} \le 1-\prod \limits _{i=1}^n \left( \frac{1-\mu _i - \nu _i}{1- \mu _i}\right) ^{\omega _i} \le \frac{-\mu _{i,\min }+\mu _{i,\max }+\nu _{i,\max }}{1-\mu _{i,\min }} \nonumber \\&\quad \Leftrightarrow -\mu _{i,\max }+\mu _{i,\min }+\nu _{i,\max } \le \frac{\gamma \left\{ 1-\prod \nolimits _{i=1}^n \left( \dfrac{1-\mu _i - \nu _i}{1- \mu _i}\right) ^{\omega _i} \right\} }{(\gamma -1)\prod \nolimits _{i=1}^n \left( \dfrac{1+(\gamma -1)\mu _i}{(\gamma -1)(1-\mu _i)}\right) ^{\omega _i} + (\gamma -1)} \le -\mu _{i,\min }+\mu _{i,\max }+c_{i,\min } \nonumber \\&\quad \Leftrightarrow \nu _{i,\max }\le \frac{\gamma \left\{ \prod \nolimits _{i=1}^n (1-\mu _i)^{\omega _i} - \prod \nolimits _{i=1}^n(1-\mu _i-\nu _i)^{\omega _i}\right\} }{\prod \nolimits _{i=1}^n (1+(\gamma -1)\mu _i)^{\omega _i} + (\gamma -1)\prod \nolimits _{i=1}^n (1-\mu _i)^{\omega _i}} \le \nu _{i,\min } \end{aligned}$$

Take $\mu _{\min } = \min \nolimits _i (\mu _{i})$, $\mu _{\max } = \max \nolimits _i (\mu _{i})$, $\nu _{\min } = \min \nolimits _i (\nu _{i})$ and $\nu _{\max } = \max \nolimits _i (\nu _{i})$. Let $IFHIWA(\alpha _1,\alpha _2,\ldots ,\alpha _n)=\alpha = \langle \mu _{\alpha },\nu _{\alpha }\rangle$ then Eqs. (3) and (4) are transformed into $\mu _{\min } \le \mu _\alpha \le \mu _{\max }, \quad \nu _{\max } \le \nu _\alpha \le \nu _{\min }$.

So, $S(\alpha ) = \mu _{\alpha } - \nu _{\alpha } \le \mu _{\max } - \nu _{\max } = S(\alpha ^+)$ and $S(\alpha ) = \mu _{\alpha } - \nu _{\alpha } \ge \mu _{\min } - \nu _{\min } = S(\alpha ^-)$. If $S(\alpha ) < S(\alpha ^+)$ and $S(\alpha ) > S(\alpha ^-)$ then by order relation between two IFNs, we have

$$\begin{aligned} \alpha ^- < IFHIWA(\alpha _1,\alpha _2,\ldots ,\alpha _n) \le \alpha ^+ \end{aligned}$$

$$\square$$

#### **Property 3**

(Monotonicity) *If*$\alpha _i$*and*$\beta _i$*,*$(i=1,2,\ldots ,n)$*be two collections of IFNs such that*$\alpha _i \le \beta _i$*for alli, then*

$$\begin{aligned} IFHIWA(\alpha _1,\alpha _2,\ldots ,\alpha _n) \le IFHIWA(\beta _1,\beta _2,\ldots ,\beta _n) \end{aligned}$$

#### *Proof*

Proof of this property is similar to above, so we omit here. $\square$

#### **Property 4**

(Shift-invariance) *If*$\beta =\langle \mu _{\beta }, \nu _{\beta }\rangle$*be another IFN, then*

$$\begin{aligned} IFHIWA(\alpha _1\oplus \beta , \alpha _2\oplus \beta , \ldots , \alpha _n\oplus \beta ) = IFHIWA(\alpha _1,\alpha _2\ldots ,\alpha _n)\oplus \beta \end{aligned}$$

#### *Proof*

As $$\alpha _i,\beta$$$$\in$$ IFNs, so

$$\begin{aligned} \alpha _i \oplus \beta = \left\langle \frac{(1+(\gamma -1)\mu _i)(1+(\gamma -1)\mu _{\beta })-(1-\mu _i)(1-\mu _{\beta })}{(1+(\gamma -1)\mu _i)(1+(\gamma -1)\mu _{\beta })+ (\gamma -1)(1-\mu _i)(1-\mu _{\beta })}, \frac{\gamma \left[ (1-\mu _i)(1-\mu _{\beta })-(1-\mu _i-\nu _i)(1-\mu _{\beta }-\nu _{\beta }) \right] }{(1+(\gamma -1)\mu _i)(1+(\gamma -1)\mu _{\beta })+(\gamma -1)(1-\mu _i)(1-\mu _{\beta })} \right\rangle \end{aligned}$$

Therefore,

$$\begin{aligned}&IFHIWA(\alpha _1\oplus \beta , \alpha _2\oplus \beta , \ldots , \alpha _n \oplus \beta )\\&\quad = \left\langle \frac{\prod \nolimits _{i=1}^n ((1+(\gamma -1)\mu _i)(1+(\gamma -1)\mu _{\beta }))^{\omega _i} -\prod \nolimits _{i=1}^n ((1-\mu _i)(1-\mu _{\beta }))^{\omega _i}}{\prod \nolimits _{i=1}^n ((1+(\gamma -1)\mu _i)(1+(\gamma -1)\mu _{\beta }))^{\omega _i} + (\gamma -1)\prod \nolimits _{i=1}^n ((1-\mu _i)(1-\mu _{\beta }))^{\omega _i}}, \right. \\&\qquad \left. \frac{\gamma \left\{ \prod \nolimits _{i=1}^n ((1-\mu _i)(1-\mu _{\beta }))^{\omega _i} -\prod \nolimits _{i=1}^n ((1-\mu _i-\nu _i)(1-\mu _{\beta }-\nu _{\beta }))^{\omega _i}\right\} }{\prod \nolimits _{i=1}^n ((1+(\gamma -1)\mu _i)(1+(\gamma -1)\mu _{\beta }))^{\omega _i} +(\gamma -1)\prod \nolimits _{i=1}^n ((1-\mu _i)(1-\mu _{\beta }))^{\omega _i}} \right\rangle \\&\quad = \left\langle \frac{\prod \nolimits _{i=1}^n ((1+(\gamma -1)\mu _i))^{\omega _i}(1+(\gamma -1)\mu _{\beta })^{\omega _i} -\prod \nolimits _{i=1}^n ((1-\mu _i))^{\omega _i}(1-\mu _{\beta })^{\omega _i}}{\prod \nolimits _{i=1}^n (1+(\gamma -1)\mu _i)^{\omega _i}(1+(\gamma -1)\mu _{\beta })^{\omega _i} + (\gamma -1) \prod \nolimits _{i=1}^n (1-\mu _i)^{\omega _i}(1-\mu _{\beta })^{\omega _i}},\right. \\&\qquad \left. \frac{\gamma \left\{ \prod \nolimits _{i=1}^n (1-\mu _i)^{\omega _i}(1-\mu _{\beta })^{\omega _i} -\prod \nolimits _{i=1}^n (1-\mu _i-\nu _i)^{\omega _i}(1-\mu _{\beta }-\nu _{\beta })^{\omega _i}\right\} }{\prod \nolimits _{i=1}^n (1+(\gamma -1)\mu _i)^{\omega _i}(1+(\gamma -1)\mu _{\beta })^{\omega _i} + (\gamma -1)\prod \nolimits _{i=1}^n (1-\mu _i)^{\omega _i}(1-\mu _{\beta })^{\omega _i}} \right\rangle \\&\quad = \left\langle \frac{\left\{ \prod \nolimits _{i=1}^n (1+(\gamma -1)\mu _i)^{\omega _i}\right\} (1+(\gamma -1)\mu _{\beta }) -\left\{ \prod \nolimits _{i=1}^n (1-\mu _i)^{\omega _i}\right\} (1-\mu _{\beta })}{\left\{ \prod \nolimits _{i=1}^n (1+(\gamma -1)\mu _i)^{\omega _i}\right\} (1+(\gamma -1)\mu _{\beta }) + (\gamma -1)\left\{ \prod \nolimits _{i=1}^n (1-\mu _i)^{\omega _i}\right\} (1-\mu _{\beta })}, \right. \\&\qquad \left. \frac{\gamma \left( \left\{ \prod \nolimits _{i=1}^n (1-\mu _i)^{\omega _i}\right\} (1-\mu _{\beta }) -\left\{ \prod \nolimits _{i=1}^n (1-\mu _i-\nu _i)^{\omega _i}\right\} (1-\mu _{\beta }-\nu _{\beta })\right) }{\left\{ \prod \nolimits _{i=1}^n (1+(\gamma -1)\mu _i)^{\omega _i}\right\} (1+(\gamma -1)\mu _{\beta }) + (\gamma -1) \left\{ \prod \nolimits _{i=1}^n (1-\mu _i)^{\omega _i}\right\} (1-\mu _{\beta })} \right\rangle \\&\quad = IFHIWA(\alpha _1,\alpha _2\ldots , \alpha _n) \oplus \beta \end{aligned}$$

$$\square$$

#### **Property 5**

(Homogeneity) *If*$\beta >0$*be a real number, then*

$$\begin{aligned} IFHIWA(\beta \alpha _1, \beta \alpha _2, \ldots , \beta \alpha _n) = \beta \,IFHIWA(\alpha _1,\alpha _2\ldots ,\alpha _n) \end{aligned}$$

#### *Proof*

Since $\alpha _i = \langle \mu _i, \nu _i \rangle$ be an IFNs for $i=1,2,\ldots ,n$. Therefore, for $\beta >0$, we have

$$\begin{aligned} \beta \alpha _i = \left\langle \frac{(1+(\gamma -1)\mu _i)^\beta -(1-\mu _i)^{\beta }}{(1+(\gamma -1)\mu _i)^{\beta }+(\gamma -1)(1-\mu _i)^{\beta }}, \frac{\gamma \left[ (1-\mu _i)^{\beta }-(1-\mu _i-\nu _i)^{\beta }\right] }{(1+(\gamma -1)\mu _i)^{\beta }+(\gamma -1)(1-\mu _i)^{\beta }} \right\rangle \end{aligned}$$

Therefore,

$$\begin{aligned}&IFHIWA(\beta \alpha _1, \beta \alpha _2, \ldots , \beta \alpha _n) \\&\quad = \left\langle \frac{\prod \nolimits _{i=1}^n [(1+(\gamma -1)\mu _i)^{\beta }]^{\omega _i} - \prod \nolimits _{i=1}^n [(1-\mu _i)^{\beta }]^{\omega _i}}{\prod \nolimits _{i=1}^n [(1+(\gamma -1)\mu _i)^{\beta }]^{\omega _i} + (\gamma -1)\prod \nolimits _{i=1}^n [(1-\mu _i)^{\beta }]^{\omega _i}}, \frac{\gamma \left\{ \prod \nolimits _{i=1}^n [(1-\mu _i)^{\beta }]^{\omega _i} - \prod \nolimits _{i=1}^n [(1-\mu _i - \nu _i)^{\beta }]^{\omega _i}\right\} }{\prod \nolimits _{i=1}^n [(1+(\gamma -1)\mu _i)^{\beta }]^{\omega _i} + (\gamma -1)\prod \nolimits _{i=1}^n [(1-\mu _i)^{\beta }]^{\omega _i}} \right\rangle \\&\quad = \left\langle \frac{\left( \prod \nolimits _{i=1}^n (1+(\gamma -1)\mu _i)^{\omega _i}\right) ^{\beta } - \left( \prod \nolimits _{i=1}^n (1-\mu _i)^{\omega _i}\right) ^{\beta }}{\left( \prod \nolimits _{i=1}^n (1+(\gamma -1)\mu _i)^{\omega _i}\right) ^{\beta } + (\gamma -1)\left( \prod \nolimits _{i=1}^n (1-\mu _i)^{\omega _i}\right) ^{\beta }}, \frac{\gamma \left\{ \left( \prod \nolimits _{i=1}^n (1-\mu _i)^{\omega _i}\right) ^{\beta } - \left( \prod \nolimits _{i=1}^n (1-\mu _i - \nu _i)^{\omega _i}\right) ^{\beta }\right\} }{\left( \prod \nolimits _{i=1}^n (1+(\gamma -1)\mu _i)^{\omega _i}\right) ^{\beta } + (\gamma -1) \left( \prod \nolimits _{i=1}^n (1-\mu _i)^{\omega _i}\right) ^{\beta }} \right\rangle \\&\quad = \beta \left\langle \frac{\prod \nolimits _{i=1}^n (1+(\gamma -1)\mu _i)^{\omega _i} - \prod \nolimits _{i=1}^n(1-\mu _i)^{\omega _i}}{\prod \nolimits _{i=1}^n(1+(\gamma -1)\mu _i)^{\omega _i} + (\gamma -1)\prod \nolimits _{i=1}^n (1-\mu _i)^{\omega _i}}, \frac{\gamma \left\{ \prod \nolimits _{i=1}^n (1-\mu _i)^{\omega _i} - \prod \nolimits _{i=1}^n (1-\mu _i-\nu _i)^{\omega _i}\right\} }{\prod \nolimits _{i=1}^n(1+(\gamma -1)\mu _i)^{\omega _i} + (\gamma -1)\prod \nolimits _{i=1}^n (1-\mu _i)^{\omega _i}} \right\rangle \\&\quad = \beta \,IFHIWA(\alpha _1,\alpha _2,\ldots ,\alpha _n) \end{aligned}$$

Hence,

$$\begin{aligned} IFHIWA(\beta \alpha _1, \ldots , \beta \alpha _n) = \beta \,IFHIWA(\alpha _1,\ldots ,\alpha _n) \end{aligned}$$

$$\square$$

#### **Property 6**

*Let*$\alpha _i=\langle \mu _{\alpha _i}, \nu _{\alpha _i}\rangle$*and*$\beta =\langle \mu _{\beta _i}, \nu _{\beta _i}\rangle (i=1,2,\ldots ,n)$*be two collections of IFNs , then*

$$\begin{aligned} IFHIWA(\alpha _1\oplus \beta _1, \alpha _2\oplus \beta _2, \ldots , \alpha _n\oplus \beta _n) = IFHIWA(\alpha _1,\alpha _2\ldots ,\alpha _n) \oplus IFHIWA(\beta _1,\beta _2\ldots ,\beta _n) \end{aligned}$$

#### *Proof*

As $\alpha _i=\langle \mu _{\alpha _i}, \nu _{\alpha _i}\rangle$ and $\beta =\langle \mu _{\beta _i}, \nu _{\beta _i}\rangle (i=1,2,\ldots ,n)$ be two collections of IFNs, then

$$\begin{aligned}&\alpha _i \oplus \beta _i = \left\langle \frac{(1+(\gamma -1)\mu _{\alpha _i})(1+(\gamma -1)\mu _{\beta _i})-(1-\mu _{\alpha _i})(1-\mu _{\beta _i})}{(1+(\gamma -1)\mu _{\alpha _i})(1+(\gamma -1) \mu _{\beta _i}) + (\gamma -1)(1-\mu _{\alpha _i})(1-\mu _{\beta _i})},\right. \\&\qquad \qquad \qquad \left. \frac{\gamma \left\{ (1-\mu _{\alpha _i})(1-\mu _{\beta _i})- (1-\mu _{\alpha _i}-\nu _{\alpha _i})(1-\mu _{\beta _i}-\nu _{\beta _i}) \right\} }{(1+(\gamma -1)\mu _{\alpha _i})(1+(\gamma -1)\mu _{\beta _i})+(\gamma -1)(1-\mu _{\alpha _i})(1-\mu _{\beta _i})} \right\rangle \end{aligned}$$

Therefore,

$$\begin{aligned}&IFHIWA(\alpha _1\oplus \beta _1, \alpha _2\oplus \beta _2, \ldots , \alpha _n\oplus \beta _n) \\&\quad = \left\langle \frac{\prod \nolimits _{i=1}^n [(1+(\gamma -1)\mu _{\alpha _i})(1+(\gamma -1)\mu _{\beta _i})]^{\omega _i} - \prod \nolimits _{i=1}^n [(1-\mu _{\alpha _i})(1-\mu _{\beta _i})]^{\omega _i}}{\prod \nolimits _{i=1}^n [(1+(\gamma -1)\mu _{\alpha _i})(1+(\gamma -1)\mu _{\beta _i})]^{\omega _i} + (\gamma -1) \prod \nolimits _{i=1}^n [(1-\mu _{\alpha _i})(1-\mu _{\beta _i})]^{\omega _i}} ,\right. \\&\qquad \qquad \left. \frac{\gamma \left\{ \prod \nolimits _{i=1}^n [(1-\mu _{\alpha _i})(1-\mu _{\beta _i})]^{\omega _i} - \prod \nolimits _{i=1}^n [(1-\mu _{\alpha _i}-\nu _{\alpha _i})(1-\mu _{\beta _i}-\nu _{\beta _i})]^{\omega _i}\right\} }{\prod \nolimits _{i=1}^n [(1+(\gamma -1)\mu _{\alpha _i})(1+(\gamma -1)\mu _{\beta _i})]^{\omega _i} + (\gamma -1)\prod \nolimits _{i=1}^n [(1-\mu _{\alpha _i})(1-\mu _{\beta _i})]^{\omega _i}} \right\rangle \\&\quad = \left\langle \frac{\prod \nolimits _{i=1}^n (1+(\gamma -1)\mu _{\alpha _i})^{\omega _i} \prod \nolimits _{i=1}^n (1+(\gamma -1)\mu _{\beta _i}){\omega _i} - \prod \nolimits _{i=1}^n (1-\mu _{\alpha _i})^{\omega _i} \prod \nolimits _{i=1}^n (1-\mu _{\beta _i})^{\omega _i}}{\prod \nolimits _{i=1}^n (1+(\gamma -1)\mu _{\alpha _i})^{\omega _i} \prod \nolimits _{i=1}^n (1+(\gamma -1)\mu _{\beta _i})^{\omega _i} + (\gamma -1)\prod \nolimits _{i=1}^n (1-\mu _{\alpha _i})^{\omega _i} \prod \nolimits _{i=1}^n (1-\mu _{\beta _i})^{\omega _i}} ,\right. \\&\qquad \qquad \left. \frac{\gamma \left\{ \prod \nolimits _{i=1}^n (1-\mu _{\alpha _i})^{\omega _i} \prod \nolimits _{i=1}^n(1-\mu _{\beta _i})^{\omega _i} - \prod \nolimits _{i=1}^n (1-\mu _{\alpha _i}-\nu _{\alpha _i})^{\omega _i}\prod \nolimits _{i=1}^n(1-\mu _{\beta _i}-\nu _{\beta _i})^{\omega _i}\right\} }{\prod \nolimits _{i=1}^n (1+(\gamma -1)\mu _{\alpha _i})^{\omega _i}\prod \nolimits _{i=1}^n(1+(\gamma -1)\mu _{\beta _i})^{\omega _i} + (\gamma -1)\prod \nolimits _{i=1}^n (1-\mu _{\alpha _i})^{\omega _i}\prod \nolimits _{i=1}^n(1-\mu _{\beta _i})^{\omega _i}} \right\rangle \\&\quad = \left\langle \frac{\prod \nolimits _{i=1}^n (1+(\gamma -1)\mu _{\alpha _i})^{\omega _i} - \prod \nolimits _{i=1}^n(1-\mu _{\alpha _i})^{\omega _i}}{\prod \nolimits _{i=1}^n(1+(\gamma -1)\mu _{\alpha _i})^{\omega _i} + (\gamma -1)\prod \nolimits _{i=1}^n (1-\mu _{\alpha _i})^{\omega _i}}, \frac{\gamma \left\{ \prod \nolimits _{i=1}^n (1-\mu _{\alpha _i})^{\omega _i} - \prod \nolimits _{i=1}^n (1-\mu _{\alpha _i}-\nu _{\alpha _i})^{\omega _i}\right\} }{\prod \limits _{i=1}^n(1+(\gamma -1)\mu _{\alpha _i})^{\omega _i} + (\gamma -1)\prod \limits _{i=1}^n (1-\mu _{\alpha _i})^{\omega _i}} \right\rangle \\&\quad \oplus \left\langle \frac{\prod \nolimits _{i=1}^n (1+(\gamma -1)\mu _{\beta _i})^{\omega _i} - \prod \nolimits _{i=1}^n(1-\mu _{\beta _i})^{\omega _i}}{\prod \nolimits _{i=1}^n(1+(\gamma -1)\mu _{\beta _i})^{\omega _i} + (\gamma -1)\prod \nolimits _{i=1}^n (1-\mu _{\beta _i})^{\omega _i}},\frac{\gamma \left\{ \prod \nolimits _{i=1}^n (1-\mu _{\beta _i})^{\omega _i} - \prod \nolimits _{i=1}^n (1-\mu _{\beta _i}-\nu _{\beta _i})^{\omega _i}\right\} }{\prod \nolimits _{i=1}^n(1+(\gamma -1)\mu _{\beta _i})^{\omega _i} + (\gamma -1)\prod \nolimits _{i=1}^n (1-\mu _{\beta _i})^{\omega _i}} \right\rangle \\&\quad = IFHIWA(\alpha _1,\alpha _2\ldots ,\alpha _n) \oplus IFHIWA(\beta _1,\beta _2\ldots ,\beta _n) \end{aligned}$$

Hence,

$$\begin{aligned} IFHIWA(\alpha _1\oplus \beta _1, \alpha _2\oplus \beta _2, \ldots , \alpha _n\oplus \beta _n) = IFHIWA(\alpha _1,\alpha _2\ldots ,\alpha _n) \oplus IFHIWA(\beta _1,\beta _2\ldots ,\beta _n) \end{aligned}$$

$$\square$$

#### **Property 7**

*Let*$\alpha _i=\langle \mu _i, \nu _i\rangle (i=1,2,\ldots ,n)$*,*$\beta = \langle \mu _{\beta }, \nu _{\beta }\rangle$*be an IFNs and If*$\eta >0$*be any real number, then*

$$\begin{aligned} IFHIWA(\eta \alpha _1\oplus \beta , \eta \alpha _2\oplus \beta , \ldots , \eta \alpha _n\oplus \beta ) = \eta \, IFHIWA(\alpha _1,\alpha _2\ldots ,\alpha _n) \oplus \beta \end{aligned}$$

#### *Proof*

By using the Properties 1, 5 and 6, we get the required proof, so it is omitted here. $\square$

### Ordered weighted averaging operator

#### **Definition 3**

Suppose there is a family of IFNs $\alpha _i=\langle \mu _i, \nu _i\rangle$ for $i=1,2,\ldots ,n$ and $IFHIOWA : \varOmega ^n \longrightarrow \varOmega$, if

$$\begin{aligned} IFHIOWA(\alpha _1,\ldots ,\alpha _n)= \omega _1\alpha _{\delta (1)} \oplus \cdots \oplus \omega _n \alpha _{\delta (n)} \end{aligned}$$

where $\omega =(\omega _1,\omega _2\ldots ,\omega _n)^T$ is the weight vector associated with IFHIOWA, $(\delta (1), \delta (2),\ldots ,\delta (n))$ is a permutation of $(1,2,3,\ldots ,n)$ such that $\alpha _{\delta (i-1)} \ge \alpha _{\delta (i)}$ for any *i*. Then IFHIOWA is called intuitionistic fuzzy Hamacher interactive ordered weighted averaging operator.

#### **Theorem 3**

*Let*$\alpha _i=\langle \mu _i,\nu _i\rangle , (i=1,2,\ldots ,n)$*be the collection of IFNs, then based on IFHIOWA operator, the aggregated IFN can be expressed as*

5 $$\begin{aligned}&IFHIOWA(\alpha _1,\alpha _2,\ldots ,\alpha _n) \nonumber \\&\quad =\left\langle \dfrac{\prod \nolimits _{i=1}^n (1+(\gamma -1)\mu _{\delta (i)})^{\omega _i}-\prod \nolimits _{i=1}^n (1-\mu _{\delta (i)})^{\omega _i}}{\prod \nolimits _{i=1}^n (1+(\gamma -1)\mu _{\delta (i)})^{\omega _i} + (\gamma -1)\prod \nolimits _{i=1}^n (1-\mu _{\delta (i)})^{\omega _i}}, \dfrac{\gamma \{\prod \nolimits _{i=1}^n (1-\mu _{\delta (i)})^{\omega _i}-\prod \nolimits _{i=1}^n (1-\mu _{\delta (i)}-\nu _{\delta (i)})^{\omega _i}\}}{\prod \nolimits _{i=1}^n (1+(\gamma -1)\mu _{\delta (i)})^{\omega _i}+(\gamma -1)\prod \nolimits _{i=1}^n (1-\mu _{\delta (i)})^{\omega _i}} \right\rangle \end{aligned}$$

Especially, $\nu _{i} = 1-\mu _{i}$ for $i=1,2,\ldots ,n$ i.e all $\alpha _i$ are reduced to $\mu _{i}$, respectively then Eq. (5) is reduced to the following form

$$\begin{aligned}&IFHIOWA(\alpha _1,\alpha _2,\ldots ,\alpha _n) \nonumber \\&\quad =\left\langle \dfrac{\prod \nolimits _{i=1}^n (1+(\gamma -1)\mu _{\delta (i)})^{\omega _i}-\prod \nolimits _{i=1}^n (1-\mu _{\delta (i)})^{\omega _i}}{\prod \nolimits _{i=1}^n (1+(\gamma -1)\mu _{\delta (i)})^{\omega _i} + (\gamma -1)\prod \nolimits _{i=1}^n (1-\mu _{\delta (i)})^{\omega _i}}, 1- \dfrac{\prod \nolimits _{i=1}^n (1+(\gamma -1)\mu _{\delta (i)})^{\omega _i}-\prod \nolimits _{i=1}^n (1-\mu _{\delta (i)})^{\omega _i}}{\prod \nolimits _{i=1}^n (1+(\gamma -1)\mu _{\delta (i)})^{\omega _i} + (\gamma -1) \prod \nolimits _{i=1}^n (1-\mu _{\delta (i)})^{\omega _i}}\right\rangle \end{aligned}$$

which becomes a fuzzy OWA operator of dimension *n* to aggregate fuzzy information.

#### *Proof*

The proof is similar to Theorem 1. $\square$

#### **Corollary 2**

*The IFHIOWA operator and IFHOWA operator have the following relation for a collections of IFNs*$\alpha _i (i=1,2,\ldots ,n)$

$$\begin{aligned} IFHIOWA(\alpha _1,\ldots ,\alpha _n) \le IFHOWA(\alpha _1,\ldots ,\alpha _n) \end{aligned}$$

As similar to those of the IFHIWA operator, the IFHIOWA operator has some properties as follows.

#### **Property 8**

*Let*$\alpha _i=\langle \mu _{i},\nu _{i}\rangle (i=1,2,\ldots ,n)$*be a collection of IFNs and*$\omega = (\omega _1,\omega _2,\ldots ,\omega _n)^T$*be the weighting vector associated with IFHIOWA operator,*$\omega _i \in [0,1], i=1,2,\ldots ,n$*and*$\sum \nolimits _{i=1}^n \omega _i = 1$*then we have the following.*

(i) *Idempotency: If all*$\alpha _i$*are equal i.e.,*$\alpha _i = \alpha$*for alli, then*$IFHIOWA(\alpha _1,\ldots ,\alpha _n) = \alpha$
(ii) *Boundedness: *
  $$\begin{aligned} \alpha _{\min } \le IFHIOWA(\alpha _1,\alpha _2,\ldots ,\alpha _n) \le \alpha _{\max } \end{aligned}$$
  *where*$$\alpha _{\min }=\min \{\alpha _1,\alpha _2,\ldots ,\alpha _n\}$$*and*$$\alpha _{\max } = \max \{\alpha _1,\alpha _2,\ldots ,\alpha _n\}$$
(iii) *Monotonicity: If*$\alpha _i$*and*$\beta _i$*,*$(i=1,2,\ldots ,n)$*be two IFNs such that*$\alpha _i \le \beta _i$*for alli, then*
  $$\begin{aligned} IFHIOWA(\alpha _1,\ldots ,\alpha _n) \le IFHIOWA(\beta _1,\ldots ,\beta _n) \end{aligned}$$
(iv) *Shift-invariance: If*$\beta =\langle \mu _{\beta },\nu _{\beta }\rangle$*be another IFN, then*
  $$\begin{aligned} IFHIOWA(\alpha _1\oplus \beta , \alpha _2\oplus \beta \oplus \ldots \oplus \alpha _n \oplus \beta ) = IFHIOWA(\alpha _1, \alpha _2, \ldots , \alpha _n)\oplus \beta \end{aligned}$$
(v) *Homogeneity: If *$\beta >0$*be a real number, then*
  $$\begin{aligned} IFHIOWA(\beta \alpha _1, \beta \alpha _2, \ldots , \beta \alpha _n) = \beta \,IFHIOWA(\alpha _1,\alpha _2\ldots ,\alpha _n) \end{aligned}$$

#### *Proof*

The proof is similar to IFHIWA properties, so we omit.

#### *Example 6*

Let $\alpha _1=\langle 0.22, 0.23\rangle$, $\alpha _2=\langle 0.04, 0.35\rangle$ and $\alpha _3=\langle 0.25, 0.23\rangle$ be three IFNs and $\omega =(0.25, 0.50, 0.25)^T$ be the position weighted vector then based on their score functions, we get their ordering as $\alpha _3 \ge \alpha _1 \ge \alpha _2$ and hence $\alpha _{\delta (1)}=\alpha _3$, $\alpha _{\delta (2)}=\alpha _1$ and $\alpha _{\delta (3)}=\alpha _2$. Then for different value of $\gamma$, the aggregated IFNs by the proposed and existing operators are summarized in Table 1.

**Table 1:** Comparison with IFHIOWA and existing operators

	$\gamma =1$		$\gamma =2$		$\gamma =3$	
	IFOWA (Xu 2007a)	Proposed	IFHOWA (Wang and Liu 2012)	Proposed	IFHOWA (Liu 2014)	Proposed
IFN	$\langle 0.1865, 0.2555\rangle$	$\langle 0.1865, 0.2570 \rangle$	$\langle 0.1836, 0.2561 \rangle$	$\langle 0.1836, 0.2579\rangle$	$\langle 0.1812, 0.2564\rangle$	$\langle 0.1812, 0.2586 \rangle$
Score	0.0690	−0.0705	−0.0726	−0.0743	−0.0752	−0.0774

Thus, it is clear from these results that

$$\begin{aligned} IFHIOWA(\alpha _1,\alpha _2,\alpha _3)< IFHOWA(\alpha _1,\alpha _2,\alpha _3) < IFOWA(\alpha _1,\alpha _2,\alpha _3) \end{aligned}$$

### Hybrid weighted averaging operator

#### **Definition 4**

Suppose there is a family of IFNs, $\alpha _i = \langle \mu _i, \nu _i\rangle , (i=1,2,\ldots ,n)$ and $IFHIHWA: \varOmega ^n \longrightarrow \varOmega$, if

$$\begin{aligned} IFHIHWA(\alpha _1,\ldots ,\alpha _n)= \omega _1 \dot{\alpha }_{\sigma (1)} \oplus \omega _2 \dot{\alpha }_{\sigma (2)} \oplus \cdots \oplus \omega _n \dot{\alpha }_{\sigma (n)} \end{aligned}$$

where $\omega =(\omega _1,\omega _2,\ldots ,\omega _n)^T$ is the weighted vector associated with IFHIHWA, $w=(w_1,w_2,\ldots ,w_n)$ is the weight vector of $\alpha _i$ such that $w_i \in [0,1], \sum \nolimits _{i=1}^n w_i =1$. Let $\dot{\alpha }_{i}$ is the *i*th largest of the weighted IFNs $\dot{\alpha }_i(= n w_i \alpha _i, i=1,2,\ldots ,n$), *n* is the number of IFNs and $(\sigma (1),\sigma (2),\ldots ,\sigma (n))$ is a permutation of $(1,2,\ldots ,n)$, such that $\dot{\alpha }_{\sigma (i-1)} \ge \dot{\alpha }_{\sigma (i)}$ for any *i*, then, function IFHIHWA is called intuitionistic fuzzy Hamacher interactive hybrid weighted averaging operator.

From the Definition 4, it has been concluded that

- It firstly weights the IFNs $\alpha _i$ by the associated weights $w_i (i=1,2,\ldots ,n)$ and multiplies these values by a balancing coefficient *n* and hence get the weighted IFNs $\dot{\alpha }_i = n w_i \alpha _i (i=1,2,\ldots ,n)$.
- It reorders the weighted arguments in descending order $(\dot{\alpha }_{\sigma (1)}, \dot{\alpha }_{\sigma (2)}, \ldots , \dot{\alpha }_{\sigma (n)})$, where $\dot{\alpha }_{\sigma (i)}$ is the *i*th largest of $\dot{\alpha }_i (i=1,2,\ldots ,n)$.
- It weights these ordered weighted IFNs $\dot{\alpha }_{\sigma (i)}$ by the IFHIWA weights $\omega _i (i=1,2,\ldots ,n)$ and then aggregates all these values into a collective one.

#### **Theorem 4**

*Let*$\alpha _i=\langle \mu _i,\nu _i\rangle$*be an IFNs,*$(i=1,2,\ldots ,n)$*then by IFHIHWA operator, the aggregated IFN* becomes

$$\begin{aligned}&IFHIHWA(\alpha _1,\alpha _2,\ldots ,\alpha _n) \\&\quad =\left\langle \dfrac{\prod \nolimits _{i=1}^n (1+(\gamma -1)\dot{\mu }_{\sigma (i)})^{\omega _i}-\prod \nolimits _{i=1}^n (1-\dot{\mu }_{\sigma (i)})^{\omega _i}}{\prod \nolimits _{i=1}^n (1+(\gamma -1)\dot{\mu }_{\sigma (i)})^{\omega _i} + (\gamma -1)\prod \nolimits _{i=1}^n (1-\dot{\mu }_{\sigma (i)})^{\omega _i}}, \dfrac{\gamma \{\prod \nolimits _{i=1}^n (1-\dot{\mu }_{\sigma (i)})^{\omega _i}-\prod \nolimits _{i=1}^n (1-\dot{\mu }_{\sigma (i)}-\dot{\nu }_{\sigma (i)})^{\omega _i}\}}{\prod \nolimits _{i=1}^n (1+(\gamma -1)\dot{\mu }_{\sigma (i)})^{\omega _i}+ (\gamma -1) \prod \nolimits _{i=1}^n (1-\dot{\mu }_{\sigma (i)})^{\omega _i}} \right\rangle \end{aligned}$$

The proof is similar to Theorem 1, so it is omitted here.

#### **Corollary 3**

*The IFHIHWA and IFHWA operators satisfies the following inequality*

$$\begin{aligned} IFHIHWA(\alpha _1,\alpha _2,\ldots ,\alpha _n) \le IFHWA(\alpha _1,\alpha _2,\ldots ,\alpha _n) \end{aligned}$$

*for a collections of IFNs*$\alpha _i$’s.

Similar to those of the IFHIWA and IFHIOWA operators, the IFHIHWA operator has also follows the same properties as described in Property 8.

### Decision making approach using proposed operators

MCDM problem is one of the fast and challenging method for every decision maker for finding the best alternative among the set of feasible one. For this, let $\{X_1,X_2,\ldots ,X_n\}$ be a set of *n* different alternatives which have been evaluate under the set of *m* different criteria $\{G_1,G_2,\ldots ,G_m\}$ by the decision maker(s). Assume that the decision maker(s) give their preferences in terms of IFNs $\alpha _{ij}=\langle \mu _{ij}, \nu _{ij}\rangle , (i=1,2,\ldots ,n; j=1,2,\ldots ,m)$, where $\mu _{ij}$ and $\nu _{ij}$ represents the degree that the alternative $X_i$ satisfies and doesn’t satisfies the attribute $G_j$ given by the decision maker respectively such that $0 \le \mu _{ij}, \nu _{ij} \le 1$ and $\mu _{ij} + \nu _{ij} \le 1$. Hence, MCDM problem can be concisely expressed in an intuitionistic fuzzy decision matrix $D=(\alpha _{ij})_{n\times m}=\langle \mu _{ij}, \nu _{ij}\rangle _{n\times m}$. Various steps used in the proposed methodology for MCDM are explained as follows:

Step 1: *Obtain the normalized intuitionistic fuzzy decision matrix*. In this step, if there are different types of criteria namely benefit (*B*) and cost (*C*) then we may transform the rating values of *B* into *C* by using the following normalization formula:
  6 $$\begin{aligned} r_{ij} = {\left\{ \begin{array}{ll} \alpha _{ij}^c; &{}\quad j \in B \\ \alpha _{ij}; &{}\quad j \in C \end{array}\right. } \end{aligned}$$
  where $\alpha _{ij}^c$ is the complement of $\alpha _{ij}$.
Step 2: *Aggregated assessment of alternatives*. Based on the decision matrix, as obtained from step 1, the overall aggregated value of alternative $X_i$, $(i=1,2,\ldots ,n)$ under the different choices of criteria $G_j$ is obtained by using IFHIWA or IFHIOWA or IFHIHWA operator and get the overall value $r_i$.
Step 3: *Compare each alternative:* Based on the overall assessment of each alternative $r_i$, a score value of each index are computed.
Step 4: *Ranking the alternative:* Rank the alternative $X_i (i=1,2,\ldots ,n)$ according to the descending value of their score values and hence select the most desirable alternative.

## Illustrative example

The above mentioned approach has been illustrated through a case study on multiple criteria decision making problem. For this, assume that a certain company has a sum of money and they want to invest it somewhere. After carefully looking in the market scenario they have decided to invest the money in the following three companies.

- $x_1$ is a car company,
- $x_2$ is a food company, and
- $x_3$ is a computer company.

according to the following four major criteria:

- $G_1$: The risk analysis,
- $G_2$: The growth analysis,
- $G_3$: The social-political impact analysis,
- $G_4$: The environmental impact analysis and
- $G_5$: The development of the society.

The weight vector corresponding to each criteria is given by the committee as $\omega =(0.1117, 0.2365, 0.3036, 0.2365, 0.1117)^T$. Assume that these alternatives are being assessed by the decision makers and give their preferences in the form of the IFNs. Then following are the step as followed by the proposed approach for accessing the best company.

### By IFHIWA operator

Step 1: As, it has been observed that there are different types of criteria so the preferences corresponding to each alternative $x_i$, $i=1,2,3$ w.r.t. each criteria $G_j$, $j=1,2,3,4,5$ are obtained in the form of normalized intuitionistic fuzzy decision matrix $D = (\alpha _{ij}) = \langle \mu _{ij}, \nu _{ij}\rangle _{3\times 5}, i=1,2,3; j=1,2,3,4,5$ as given below. 7
Step 2: Utilize the IFHIWA operator corresponding to $\gamma = 2$ to compute the overall assessment of each alternative as
  $$\begin{aligned} r_1 & = IFHIWA(r_{11},r_{12},r_{13},r_{14}, r_{15}) \\ & = \left\langle \frac{\begin{aligned}&(1.2)^{0.1117}(1.4)^{0.2365}(1.5)^{0.3036}(1.3)^{0.2365}(1.7)^{0.1117} - \\&(0.8)^{0.1117}(0.6)^{0.2365}(0.5)^{0.3036}(0.7)^{0.2365}(0.3)^{0.1117} \end{aligned}}{ \begin{aligned}&(1.2)^{0.1117}(1.4)^{0.2365}(1.5)^{0.3036}(1.3)^{0.2365}(1.7)^{0.1117} + \\&(0.8)^{0.1117}(0.6)^{0.2365}(0.5)^{0.3036}(0.7)^{0.2365}(0.3)^{0.1117} \end{aligned} }, \frac{\begin{aligned}&2\left\{ (0.8)^{0.1117}(0.6)^{0.2365}(0.5)^{0.3036}(0.7)^{0.2365}(0.3)^{0.1117} - \right. \\&\left. (0.3)^{0.1117}(0.4)^{0.2365}(0.1)^{0.3036}(0.4)^{0.2365}(0.2)^{0.1117}\right\} \end{aligned}}{\begin{aligned}&(1.2)^{0.1117}(1.4)^{0.2365}(1.5)^{0.3036}(1.3)^{0.2365}(1.7)^{0.1117} + \\&(0.8)^{0.1117}(0.6)^{0.2365}(0.5)^{0.3036}(0.7)^{0.2365}(0.3)^{0.1117} \end{aligned}} \right\rangle \\ & = \langle 0.4298, 0.3317\rangle \\ r_2 & = IFHIWA(r_{21},r_{22},r_{23},r_{24}, r_{25}) \\ & = \left\langle \frac{\begin{aligned}&(1.2)^{0.1117}(1.6)^{0.2365}(1.4)^{0.3036}(1.4)^{0.2365}(1.6)^{0.1117} - \\&(0.8)^{0.1117}(0.4)^{0.2365}(0.6)^{0.3036}(0.6)^{0.2365}(0.4)^{0.1117} \end{aligned}}{ \begin{aligned}&(1.2)^{0.1117}(1.6)^{0.2365}(1.4)^{0.3036}(1.4)^{0.2365}(1.6)^{0.1117} + \\&(0.8)^{0.1117}(0.4)^{0.2365}(0.6)^{0.3036}(0.6)^{0.2365}(0.4)^{0.1117} \end{aligned} }, \frac{\begin{aligned}&2\left\{ (0.8)^{0.1117}(0.4)^{0.2365}(0.6)^{0.3036}(0.6)^{0.2365}(0.4)^{0.1117} - \right. \\&\left. (0.1)^{0.1117}(0.1)^{0.2365}(0.3)^{0.3036}(0.2)^{0.2365}(0.3)^{0.1117}\right\} \end{aligned}}{\begin{aligned}&(1.2)^{0.1117}(1.6)^{0.2365}(1.4)^{0.3036}(1.4)^{0.2365}(1.6)^{0.1117} + \\&(0.8)^{0.1117}(0.4)^{0.2365}(0.6)^{0.3036}(0.6)^{0.2365}(0.4)^{0.1117} \end{aligned}} \right\rangle \\ & = \langle 0.4564, 0.3557\rangle \\ r_3 & = IFHIWA(r_{31},r_{32},r_{33},r_{34}, r_{35}) \\ & = \left\langle \frac{\begin{aligned}&(1.2)^{0.1117}(1.5)^{0.2365}(1.4)^{0.3036}(1.3)^{0.2365}(1.6)^{0.1117} - \\&(0.8)^{0.1117}(0.5)^{0.2365}(0.6)^{0.3036}(0.7)^{0.2365}(0.4)^{0.1117} \end{aligned}}{ \begin{aligned}&(1.2)^{0.1117}(1.5)^{0.2365}(1.4)^{0.3036}(1.3)^{0.2365}(1.6)^{0.1117} + \\&(0.8)^{0.1117}(0.5)^{0.2365}(0.6)^{0.3036}(0.7)^{0.2365}(0.4)^{0.1117} \end{aligned} }, \frac{\begin{aligned}&2\left\{ (0.8)^{0.1117}(0.5)^{0.2365}(0.6)^{0.3036}(0.7)^{0.2365}(0.4)^{0.1117} - \right. \\&\left. (0.1)^{0.1117}(0.2)^{0.2365}(0.1)^{0.3036}(0.3)^{0.2365}(0.2)^{0.1117}\right\} \end{aligned}}{\begin{aligned}&(1.2)^{0.1117}(1.5)^{0.2365}(1.4)^{0.3036}(1.3)^{0.2365}(1.6)^{0.1117} + \\&(0.8)^{0.1117}(0.5)^{0.2365}(0.6)^{0.3036}(0.7)^{0.2365}(0.4)^{0.1117} \end{aligned}} \right\rangle \\& = \langle 0.4068, 0.4267 \rangle \end{aligned}$$
Step 3: The scores values corresponding to each $r_i (i=1,2,3,4,5)$ is.
  $$\begin{aligned} S(r_1) = 0.0981; \quad S(r_2)= 0.1007; \quad S(r_3)= -0.0199 \end{aligned}$$
Step 4: Since $S_2>S_1>S_3$ thus we have $x_2 \succ x_1 \succ x_3$. Hence, the best financial strategy is $x_2$ i.e. to invest in the food company.

### By IFHIHWA operator

In order to aggregate these different IFNs by using IFHIHWA operator, the following steps are utilize.

Step 1: Use the normalized fuzzy decision matrix as given in Eq. (7).
Step 2: Compute the IFNs $\dot{r}_{ij} = (5w_j)r_{ij}$, where $w_j=(0.25, 0.20, 0.15, 0.18, 0.22)^T$ we get
  $$\begin{aligned}&\dot{r}_{11}= \langle 0.1206, 0.5958\rangle , \quad \dot{r}_{12}=\langle 0.4, 0.2\rangle , \quad \dot{r}_{13}= \langle 0.6098, 0.3075\rangle , \\&\dot{r}_{14}=\langle 0.3470, 0.2716\rangle , \quad \dot{r}_{15}= \langle 0.6721, 0.1099\rangle , \quad \dot{r}_{21}= \langle 0.1206, 0.7947\rangle , \\&\dot{r}_{22}=\langle 0.6, 0.3\rangle , \quad \dot{r}_{23}= \langle 0.5224, 0.2281\rangle , \quad \dot{r}_{24}=(0.4462, 0.3638\rangle , \\&\dot{r}_{25}=\langle 0.5650, 0.1099\rangle , \quad \dot{r}_{31}= \langle 0.1206, 0.7947\rangle , \quad \dot{r}_{32}= \langle 0.5, 0.3\rangle , \\&\dot{r}_{33}= \langle 0.5224, 0.3902\rangle , \quad \dot{r}_{34}=\langle 0.3470, 0.3638\rangle , \quad \dot{r}_{35}= \langle 0.5650, 0.2194\rangle \end{aligned}$$
  Now, reorders these IFNs based on their score function, and get ordered weighted IFNs $\dot{r}_{\sigma (ij)}$ as
  $$\begin{aligned}&\dot{r}_{\sigma (11)} = \langle 0.6721, 0.1099\rangle , \qquad \dot{r}_{\sigma (12)} = \langle 0.6098, 0.3075\rangle , \qquad \dot{r}_{\sigma (13)} = \langle 0.4000, 0.2000\rangle , \\&\dot{r}_{\sigma (14)} = \langle 0.3470, 0.2716\rangle , \qquad \dot{r}_{\sigma (15)} = \langle 0.1206, 0.5958\rangle , \qquad \dot{r}_{\sigma (21)} = \langle 0.5650, 0.1099\rangle , \\&\dot{r}_{\sigma (22)} = \langle 0.6000, 0.3000\rangle , \qquad \dot{r}_{\sigma (23)} = \langle 0.5224, 0.2281\rangle , \qquad \dot{r}_{\sigma (24)} = \langle 0.4462, 0.3638\rangle , \\&\dot{r}_{\sigma (25)} = \langle 0.1206, 0.7947\rangle , \qquad \dot{r}_{\sigma (31)} = \langle 0.5650, 0.2194\rangle , \qquad \dot{r}_{\sigma (32)} = \langle 0.5000, 0.3000\rangle , \\&\dot{r}_{\sigma (33)} = \langle 0.5224, 0.3902\rangle , \qquad \dot{r}_{\sigma (34)} = \langle 0.3470, 0.3638\rangle , \qquad \dot{r}_{\sigma (35)} = \langle 0.1206, 0.7947\rangle \end{aligned}$$
  Thus, finally utilize these ordered weighted IFNs and the weight vector $\omega$ corresponding to each criteria, the aggregated value have been obtained corresponding to each alternative as
  $$\begin{aligned} r_1 = \langle 0.4263, 0.2820\rangle , \quad r_2 = \langle 0.4450, 0.3574\rangle , \quad r_3 = \langle 0.4113, 0.4005 \rangle \end{aligned}$$
Step 3: The score values corresponding to above $$r_i$$$$(i=1,2,3,4)$$ is
  $$\begin{aligned} S(r_1)= 0.1443, \quad S(r_2)=0.0876, \quad S(r_3)= 0.0108 \end{aligned}$$
Step 4: Thus, $r_1\succ r_2\succ r_3$ and their corresponding alternative order are $x_1 \succ x_2 \succ x_3$. Therefore, the best company for investing the money is $x_1$ (car company).

### Comparison with the existing methodologies

#### By Xu (2007a) approach

If we utilize IFWA (Xu 2007a) operator to aggregate these IFNs then we get their corresponding aggregated values as

$$\begin{aligned} r_1 & = IFWA(r_{11}, r_{12}, r_{13}, r_{14}, r_{15}) \\ & = \left\langle 1-(0.8)^{0.1117}(0.6)^{0.2365}(0.5)^{0.3036}(0.3)^{0.2365}(0.7)^{0.1117}, (0.5)^{0.1117} (0.2)^{0.2365} (0.4)^{0.3036} (0.3)^{0.2365} (0.1)^{0.1117} \right\rangle \\ & = \langle 0.4373, 0.2785 \rangle \\ r_2 & = IFWA(r_{21}, r_{22}, r_{23}, r_{24}, r_{25}) \\& = \left\langle 1-(0.8)^{0.1117}(0.4)^{0.2365}(0.6)^{0.3036}(0.6)^{0.2365}(0.4)^{0.1117}, (0.7)^{0.1117} (0.3)^{0.2365} (0.3)^{0.3036} (0.4)^{0.2365} (0.1)^{0.1117} \right\rangle \\ & = \langle 0.4620, 0.3122 \rangle \\ r_3 & = IFWA(r_{31}, r_{32}, r_{33}, r_{34}, r_{35}) \\ & = \left\langle 1-(0.8)^{0.1117}(0.5)^{0.2365}(0.6)^{0.3036}(0.7)^{0.2365}(0.4)^{0.1117}, (0.7)^{0.1117} (0.3)^{0.2365} (0.5)^{0.3036} (0.4)^{0.2365} (0.2)^{0.1117} \right\rangle \\ & = \langle 0.4118, 0.3940 \rangle \end{aligned}$$

and hence order relation is $r_1\succ r_2 \succ r_3$ which corresponds to $x_1 \succ x_2 \succ x_3$.

#### By Wang and Liu (2012) approach

If we utilize IFEWA (Wang and Liu 2012) operator to aggregate these IFNs then we get their corresponding aggregated values

$$\begin{aligned} r_1 & = IFEWA(r_{11},r_{12},r_{13},r_{14}, r_{15}) \\ & = \left\langle \frac{\begin{aligned}&(1.2)^{0.1117}(1.4)^{0.2365}(1.5)^{0.3036}(1.3)^{0.2365}(1.7)^{0.1117} - \\&(0.8)^{0.1117}(0.6)^{0.2365}(0.5)^{0.3036}(0.7)^{0.2365}(0.3)^{0.1117} \end{aligned}}{ \begin{aligned}&(1.2)^{0.1117}(1.4)^{0.2365}(1.5)^{0.3036}(1.3)^{0.2365}(1.7)^{0.1117} + \\&(0.8)^{0.1117}(0.6)^{0.2365}(0.5)^{0.3036}(0.7)^{0.2365}(0.3)^{0.1117} \end{aligned} }, \frac{\begin{aligned}&2 (0.5)^{0.1117}(0.2)^{0.2365}(0.4)^{0.3036}(0.3)^{0.2365}(0.1)^{0.1117} \end{aligned}}{\begin{aligned}&(1.5)^{0.1117}(1.8)^{0.2365}(1.6)^{0.3036}(1.7)^{0.2365}(1.9)^{0.1117} + \\&(0.5)^{0.1117}(0.2)^{0.2365}(0.4)^{0.3036}(0.3)^{0.2365}(0.1)^{0.1117} \end{aligned}} \right\rangle \\ & = \langle 0.4298, 0.2831 \rangle \\ r_2 & = IFEWA(r_{21},r_{22},r_{23},r_{24}, r_{25}) \\ & = \left\langle \frac{\begin{aligned}&(1.2)^{0.1117}(1.6)^{0.2365}(1.4)^{0.3036}(1.4)^{0.2365}(1.6)^{0.1117} - \\&(0.8)^{0.1117}(0.4)^{0.2365}(0.6)^{0.3036}(0.6)^{0.2365}(0.4)^{0.1117} \end{aligned}}{ \begin{aligned}&(1.2)^{0.1117}(1.6)^{0.2365}(1.4)^{0.3036}(1.4)^{0.2365}(1.6)^{0.1117} + \\&(0.8)^{0.1117}(0.4)^{0.2365}(0.6)^{0.3036}(0.6)^{0.2365}(0.4)^{0.1117} \end{aligned} }, \frac{\begin{aligned}&2(0.7)^{0.1117}(0.3)^{0.2365}(0.3)^{0.3036}(0.4)^{0.2365}(0.1)^{0.1117} \end{aligned}}{\begin{aligned}&(1.3)^{0.1117}(1.7)^{0.2365}(1.7)^{0.3036}(1.6)^{0.2365}(1.9)^{0.1117} + \\&(0.7)^{0.1117}(0.3)^{0.2365}(0.3)^{0.3036}(0.4)^{0.2365}(0.1)^{0.1117} \end{aligned}} \right\rangle \\ & = \langle 0.4564, 0.3188 \rangle \\ r_3 & = IFHIWA(r_{31},r_{32},r_{33},r_{34}, r_{35}) \\ & = \left\langle \frac{\begin{aligned}&(1.2)^{0.1117}(1.5)^{0.2365}(1.4)^{0.3036}(1.3)^{0.2365}(1.6)^{0.1117} - \\&(0.8)^{0.1117}(0.5)^{0.2365}(0.6)^{0.3036}(0.7)^{0.2365}(0.4)^{0.1117} \end{aligned}}{ \begin{aligned}&(1.2)^{0.1117}(1.5)^{0.2365}(1.4)^{0.3036}(1.3)^{0.2365}(1.6)^{0.1117} + \\&(0.8)^{0.1117}(0.5)^{0.2365}(0.6)^{0.3036}(0.7)^{0.2365}(0.4)^{0.1117} \end{aligned} }, \frac{\begin{aligned}&2(0.7)^{0.1117}(0.3)^{0.2365}(0.5)^{0.3036}(0.4)^{0.2365}(0.2)^{0.1117} \end{aligned}}{\begin{aligned}&(1.3)^{0.1117}(1.7)^{0.2365}(1.5)^{0.3036}(1.6)^{0.2365}(1.8)^{0.1117} + \\&(0.7)^{0.1117}(0.3)^{0.2365}(0.5)^{0.3036}(0.4)^{0.2365}(0.2)^{0.1117} \end{aligned}} \right\rangle \\ & = \langle 0.4068, 0.4000 \rangle \end{aligned}$$

and hence ranking of these alternatives are $r_1 \succ r_2 \succ r_3$ and thus their corresponding alternative ranking order are $x_1 \succ x_2 \succ x_3$.

### Sensitivity analysis

**Fig. 1:**
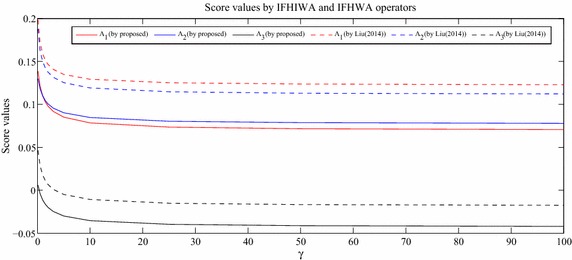
Score value versus $\gamma$ parameter by IFHIWA operator

**Fig. 2:**
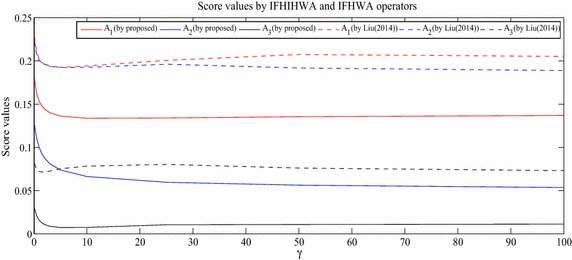
Score value versus $\gamma$ parameter by IFHIHWA operator

To analyze the effect of $\gamma$ on the most desirable alternatives on the given attributes, we use the different values of $\gamma$ in the proposed approach to rank the alternatives. The corresponding score values and their ranking order are summarized in Table 2 along with the results as obtained by Liu (2014) approach. From this table, it has been analyzed that with the increase of the parameter $\gamma$, their score values corresponding to each alternative is decrease which is in accordance with the results of as obtained from Liu (2014) approach. The variations of the ranking of these three companies with respect to the value of parameter $\gamma$ by the proposed IFHIWA and IFHIHWA operator are shown in Figs. 1 and 2 respectively. Furthermore, it has been obtained that the score value of each alternative by the proposed approach is less than the existing approach which shows the optimistic attitude nature to the decision makers’ which validates the Corollary 1.

**Table 2:** Ordering of the alternatives for different $\gamma$

$\gamma$	Approach	Score function by IFHIWA				Score function by IFHIOWA				Score function by IFHIHWA			
		$S(x_1)$	$S(x_2)$	$S(x_3)$	Ranking	$S(x_1)$	$S(x_2)$	$S(x_3)$	Ranking	$S(x_1)$	$S(x_2)$	$S(x_3)$	Ranking
0.1	Proposed	0.1386	0.1298	0.0060	$x_1 \succ x_2 \succ x_3$	0.1386	0.1298	0.0060	$x_1 \succ x_2 \succ x_3$	0.1790	0.1260	0.0294	$x_1 \succ x_2 \succ x_3$
	Liu (2014)	0.1984	0.1878	0.0464	$x_1\succ x_2\succ x_3$	0.1984	0.1878	0.0464	$x_1\succ x_2\succ x_3$	0.2280	0.2227	0.0811	$x_1\succ x_2\succ x_3$
0.5	Proposed	0.1214	0.1183	−0.0039	$x_1 \succ x_2 \succ x_3$	0.1214	0.1183	−0.0039	$x_1 \succ x_2 \succ x_3$	0.1625	0.1107	0.0211	$x_1 \succ x_2 \succ x_3$
	Liu (2014)	0.1722	0.1627	0.0288	$x_1\succ x_2\succ x_3$	0.1722	0.1627	0.0288	$x_1\succ x_2\succ x_3$	0.2105	0.2072	0.0746	$x_1\succ x_2\succ x_3$
1.0	Proposed	0.1099	0.1099	−0.0114	$x_2 \succ x_1 \succ x_3$	0.1099	0.1099	−0.0114	$x_2 \succ x_1 \succ x_3$	0.1530	0.0996	0.0157	$x_1 \succ x_2 \succ x_3$
	Liu (2014)	0.1588	0.1498	0.0178	$x_1\succ x_2\succ x_3$	0.1588	0.1498	0.0178	$x_1\succ x_2\succ x_3$	0.2023	0.2007	0.0721	$x_1\succ x_2\succ x_3$
1.5	Proposed	0.1029	0.1045	−0.0163	$x_2 \succ x_1 \succ x_3$	0.1029	0.1045	−0.0163	$x_2 \succ x_1 \succ x_3$	0.1479	0.0925	0.0127	$x_1 \succ x_2 \succ x_3$
	Liu (2014)	0.1514	0.1425	0.0112	$x_1\succ x_2\succ x_3$	0.1514	0.1425	0.0112	$x_1\succ x_2\succ x_3$	0.1982	0.1978	0.0715	$x_1\succ x_2\succ x_3$
2.0	Proposed	0.0981	0.1007	−0.0199	$x_2 \succ x_1 \succ x_3$	0.0981	0.1007	−0.0199	$x_2 \succ x_1 \succ x_3$	0.1443	0.0876	0.0108	$x_1 \succ x_2 \succ x_3$
	Liu (2014)	0.1467	0.1377	0.0068	$x_1\succ x_2\succ x_3$	0.1467	0.1377	0.0068	$x_1\succ x_2\succ x_3$	0.1960	0.1964	0.0716	$x_1\succ x_2\succ x_3$
3.0	Proposed	0.0918	0.0957	−0.0247	$x_2 \succ x_1 \succ x_3$	0.0918	0.0957	−0.0247	$x_2 \succ x_1 \succ x_3$	0.1400	0.0810	0.0087	$x_1 \succ x_2 \succ x_3$
	Liu (2014)	0.1408	0.1316	0.0011	$x_1\succ x_2\succ x_3$	0.1408	0.1316	0.0011	$x_1\succ x_2\succ x_3$	0.1937	0.1941	0.0725	$x_1\succ x_2\succ x_3$
5.0	Proposed	0.0851	0.0902	−0.0300	$x_2 \succ x_1 \succ x_3$	0.0851	0.0902	−0.0300	$x_2 \succ x_1 \succ x_3$	0.1362	0.0738	0.0073	$x_1 \succ x_2 \succ x_3$
	Liu (2014)	0.1349	0.1253	−0.0049	$x_1\succ x_2\succ x_3$	0.1349	0.1253	−0.0049	$x_1\succ x_2\succ x_3$	0.1926	0.1923	0.0753	$x_1\succ x_2\succ x_3$
10	Proposed	0.0785	0.0847	−0.0354	$x_2 \succ x_1 \succ x_3$	0.0785	0.0847	−0.0354	$x_2 \succ x_1 \succ x_3$	0.1337	0.0663	0.0074	$x_1 \succ x_2 \succ x_3$
	Liu (2014)	0.1293	0.1192	−0.0107	$x_1\succ x_2\succ x_3$	0.1293	0.1192	−0.0107	$x_1\succ x_2\succ x_3$	0.1941	0.1925	0.0783	$x_1\succ x_2\succ x_3$
25	Proposed	0.0736	0.0804	−0.0396	$x_2 \succ x_1 \succ x_3$	0.0736	0.0804	−0.0396	$x_2 \succ x_1 \succ x_3$	0.1340	0.0596	0.0105	$x_1 \succ x_2 \succ x_3$
	Liu (2014)	0.1252	0.1147	−0.0151	$x_1\succ x_2\succ x_3$	0.1252	0.1147	−0.0151	$x_1\succ x_2\succ x_3$	0.2006	0.1962	0.0803	$x_1\succ x_2\succ x_3$
50	Proposed	0.0717	0.0788	−0.0412	$x_2 \succ x_1 \succ x_3$	0.0717	0.0788	−0.0412	$x_2 \succ x_1 \succ x_3$	0.1355	0.0563	0.0108	$x_1 \succ x_2 \succ x_3$
	Liu (2014)	0.1237	0.1130	−0.0167	$x_1\succ x_2\succ x_3$	0.1237	0.1130	−0.0167	$x_1\succ x_2\succ x_3$	0.2074	0.1918	0.0761	$x_1\succ x_2\succ x_3$
100	Proposed	0.0707	0.0779	−0.0421	$x_2 \succ x_1 \succ x_3$	0.0707	0.0779	−0.0421	$x_2 \succ x_1 \succ x_3$	0.1369	0.0535	0.0112	$x_1 \succ x_2 \succ x_3$
	Liu (2014)	0.1229	0.1122	−0.0175	$x_1\succ x_2\succ x_3$	0.1229	0.1122	−0.0175	$x_1\succ x_2\succ x_3$	0.2052	0.1888	0.0731	$x_1\succ x_2\succ x_3$

## Conclusion

In this article, the objective of the work is to present some series of an averaging aggregation operators by using hamacher operations. For this, firstly shortcoming of the various existing operations and their corresponding aggregator operators is highlighted. These shortcoming has been resolved by defining a new set of operational laws on the intuitionistic fuzzy set environment by considering the degree of interaction or hesitation between the grades of functions. Based on these laws, some series of an averaging aggregation operators namely IFHIWA, IFHIOWA and IFHIHWA have been proposed. The desirable properties corresponding to each operator has been discussed. It has been observed from the operators that some existing operators IFWA and IFEWA are taken as a special case of the proposed operators. These operators have been applied to solve the MCDM problem for showing the substantiality and effectiveness of the approach. From the proposed approach, it has been concluded that it contain almost all of arithmetic aggregation operators for IFNs based on different $\gamma$ and hence proposed operators are more general and flexible.
